# Employing defined bioconjugates to generate chemically functionalised gold nanoparticles for *in vitro* diagnostic applications†

**DOI:** 10.1039/d1nr02584h

**Published:** 2021-06-22

**Authors:** Daniel A. Richards, Michael R. Thomas, Peter A. Szijj, James Foote, Yiyun Chen, João C. F. Nogueira, Vijay Chudasama, Molly M. Stevens

**Affiliations:** Department of Materials, Imperial College London London UK; Department of Bioengineering, Imperial College London UK; Institute of Biomedical Engineering, Imperial College London London UK; London Centre for Nanotechnology, University College London London UK; Department of Chemistry, University College London London UK; Department of Medical Biochemistry and Biophysics Stockholm Sweden m.stevens@imperial.ac.uk; Department of Biochemical Engineering, University College London WC1E 6BT London UK

## Abstract

Novel methods for introducing chemical and biological functionality to the surface of gold nanoparticles serve to increase the utility of this class of nanomaterials across a range of applications. To date, methods for functionalising gold surfaces have relied upon uncontrollable non-specific adsorption, bespoke chemical linkers, or non-generalisable protein–protein interactions. Herein we report a versatile method for introducing functionality to gold nanoparticles by exploiting the strong interaction between chemically functionalised bovine serum albumin (*f*-BSA) and citrate-capped gold nanoparticles (AuNPs). We establish the generalisability of the method by introducing a variety of functionalities to gold nanoparticles using cheap, commercially available chemical linkers. The utility of this approach is further demonstrated through the conjugation of the monoclonal antibody Ontruzant to *f*-BSA–AuNPs using inverse electron-demand Diels–Alder (iEDDA) click chemistry, a hitherto unexplored chemistry for AuNP–IgG conjugation. Finally, we show that the AuNP–Ontruzant particles generated *via f*-BSA–AuNPs have a greater affinity for their target in a lateral flow format when compared to conventional physisorption, highlighting the potential of this technology for producing sensitive diagnostic tests.

## Abstract 2

Herein we report a versatile method for introducing functionality to gold nanoparticles by exploiting the strong interaction between chemically functionalised bovine serum albumin (*f*-BSA) and citrate-capped gold nanoparticles (AuNPs).

## Graphical abstract

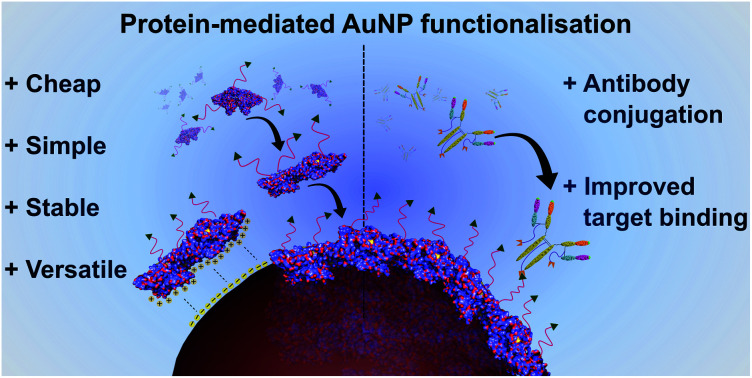

## Introduction

Over the last few decades nanomaterials have transitioned from academic curiosities to established research tools, finding use in applications ranging from clinically approved therapeutics and diagnostics,^1–4^ through to emerging technologies such as tissue engineering and bioelectronics.^5,6^ This high rate of adoption is a result of their unique optical, thermal, plasmonic, and electrical properties. Whilst the intrinsic properties of nanomaterials are powerful in their own right, it is often desirable to improve or complement them through the attachment of chemical or biological moieties. The additional functionality granted by surface modification has enabled the use of nanomaterials in an ever-increasing array of technologies.^7^

Of the many established classes of nanomaterials, gold nanoparticles (AuNPs) remain one of the most popular. The unique plasmonic properties of colloidal gold instil gold nanoparticles with remarkable optical density, allowing them to be visible with the naked eye at even sub-picomolar concentrations. This makes them ideal for both *in vivo* and *in vitro* diagnostics, and has led to a plethora of downstream nanomedical applications *e.g.*, photothermal therapy.^8^ Gold nanoparticles are also easy to produce, even on a large scale,^9^ for a relatively low cost. However, gold nanoparticles also display many properties that have hindered their use for certain applications. The surface of gold is highly reactive and prone to fouling by both chemical and biological agents; this can alter the desirable properties, and ultimately lead to aggregation.^10^ Fortunately, these hindrances can be minimised through surface modification of gold nanoparticles *via* chemical or biological methods.^11,12^

Due to the ubiquitous nature of gold nanoparticles, many methods for modifying their surfaces have been developed.^13^ The most common approach has been to exploit the strong Au–S bond between gold surfaces and thiols to introduce heterobifunctional chemical linkers. These linkers are frequently pegylated to improve aqueous solubility and also add some protection against fouling. Whilst this method is reliable and can be achieved using cheap, commercially available reagents, the reliance on thiol chemistry precludes the installation of thiol-sensitive chemical functionalities. Other approaches have exploited the innate reactivity between gold surfaces and proteins to introduce functionality. This approach has been effectively employed to install anchoring proteins such as streptavidin,^14^ protein A/G,^15–17^ and SpyCapture^18^ onto gold surfaces. In addition to increasing the functionality of gold nanoparticles, these protein-based systems offer benefit through passivation of the gold surface, helping to guard against fouling and particle aggregation.^18^ Whilst these methods are effective, they are not without limitations. The streptavidin–biotin bond, though strong, is non-covalent and subject to dissociation under certain conditions. Additionally, affinity systems such as protein A/G–IgG and SpyCapture–SpyTag only facilitate conjugation to entities containing the corresponding protein-based affinity partner. In many cases, these affinity-based methods necessitate time consuming protein engineering which can limit the uptake of this technology.

Herein we report a system that combines the flexibility and versatility of chemical functionalisation, with the additional stability provided by protein-based surface assembly. We have achieved this by first chemically modifying an idealized protein with desirable functionality, and subsequently depositing the protein onto the gold surface. The protein acts as a sacrificial scaffold for the chemical functionality whilst simultaneously providing surface passivation. To provide sufficient benefit over existing methods, an ideal carrier protein needed to satisfy several criteria:

(1) Compatible with a wide variety of chemical functionalities, with no significant side-reactions;

(2) Affordable, and can be easily modified to a high degree using cheap, commercially available reagents;

(3) Easily deposited onto gold surfaces under mild conditions that are compatible with a broad range of chemical functionalities;

(4) Forms a strong and stable interaction with gold surfaces, without interfering with desirable particle properties;

(5) The resulting functionalised gold nanoparticles should remain stable over practical time-scales and under challenging conditions.

With these criteria in mind, we selected bovine serum albumin (BSA) as a carrier protein. Albumin, the most abundant protein in blood, is cheap, has an abundance of nucleophilic amino acid side chains for chemical conjugation, and has a high affinity for gold surfaces.^19^ Albumin is also frequently used to stabilize gold nanoparticles, particularly for *in vitro* diagnostic applications,^20^ so a wealth of information on the interactions between gold nanoparticles and albumin exists in the literature.^20,21^

**Scheme 1:**
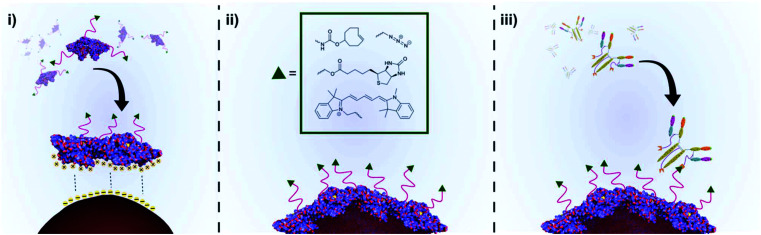
An illustration of the preparation of functional BSA coated AuNPs and subsequent conjugation to a disease-specific IgG. (i) Chemically functionalised BSA (*f*-BSA) approaches the surface of citrate capped AuNPs. Conformational changes in the structure of *f*-BSA as it interacts with the AuNP surface lead to unfolding of the protein, whilst non-covalent interactions between the protein and AuNP surface lead to a stable protein corona. (ii) A layer of *f*-BSA on the gold surface leads to stable, chemically functionalised gold nanoparticles. (iii) Chemical functionality can facilitate downstream applications such as conjugation of disease-targeting antibodies.

Our albumin-based system combines the benefits of heterobifunctional chemical linkers with the stability afforded by gold nanoparticle–albumin complexes to generate chemically functionalised gold nanoparticles. We demonstrate that by exploiting the strong interaction between chemically functionalised BSA (*f*-BSA) and gold surfaces, we can prepare functionalised gold nanoparticles that remain stable in complex biological media. This highly generalisable system enables the introduction of multiple unique chemical functionalities and allows for delicate control over the degree of functionalisation. Additionally, we demonstrate that this system can be used to assemble an antibody layer onto *f*-BSA coated gold nanoparticles using simple chemistries (Scheme 1).

## Results and discussion

### Preparation of functionalised BSA

**Fig. 1:**
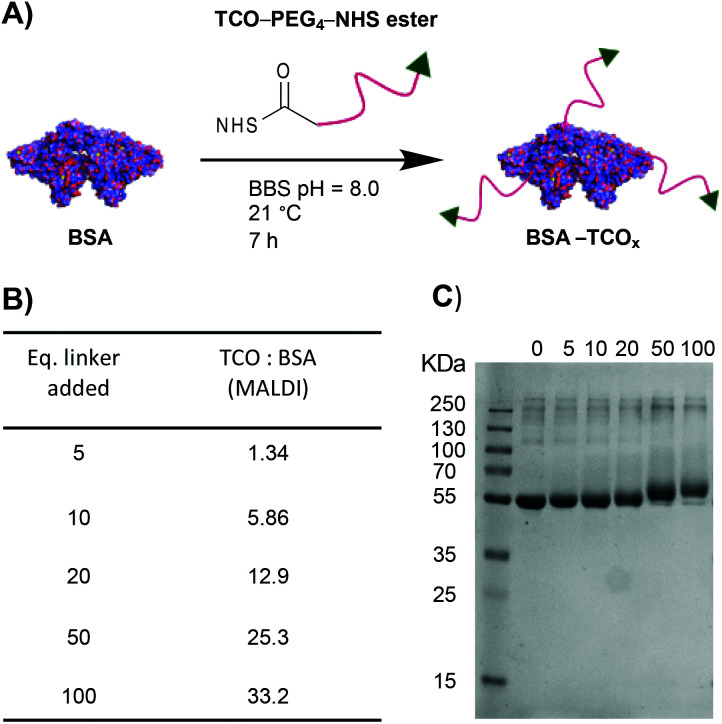
(A) Schematic representation of the modification of BSA with TCO–PEG_4_. (B) MALDI data showing the average number of TCO–PEG_4_ molecules per BSA; for full MALDI spectra and details of the calculations see Fig. S1.† (C) SDS-PAGE gel of BSA incubated with various equivalents of TCO–PEG_4_ (0 = native BSA).

To test the premise that functionalised BSA is capable of adhering to the surface of gold nanoparticles, we designed a robust method for modifying the protein with an appropriate functional group. We chose the electron-deficient *trans*-cyclooctene (TCO) moiety as a model, due to its ability to engage in rapid, biorthogonal, catalyst-free inverse electron demand Diels–Alder reactions (iEDDA) with tetrazines.^22^ iEDDA “click” reactions are the premier tool for the facile conjugation of small molecules to biomolecules, and are increasingly being employed for attaching biomolecules to surfaces.^22,23^ Thus, incorporation of this functionality maximizes the downstream applications of the resulting BSA–AuNP complexes. Functionalisation of BSA with TCO was accomplished *via* reaction of the lysine residues present on the protein's surface with a heterobifunctional TCO–PEG_4_–*N*-hydroxysuccinimide (TCO–PEG_4_–NHS) linker (Fig. 1A). We incubated native BSA with 5–100 equiv. of TCO–PEG_4_–NHS, and assessed the resulting bioconjugates by SDS-PAGE, UV-Vis spectrometry, and matrix-assisted laser-directed ionization (MALDI) mass spectrometry (Fig. 1B, C and Fig. S1†).

The choice of chemistry was driven by the desire to maximize the degree of functionalisation by targeting the abundance (60 residues) of lysine residues present on BSA. Though the use of site-selective chemistries (*e.g.*, cysteine-selective) would provide a more homogenous product, the lack of readily available reaction sites drastically reduces the potential degree of functionalisation.^24^ The optimised protocol granted BSA with TCO : BSA ratios of between approximately 1 : 1 and 33 : 1, as determined by MALDI mass spectrometry (Fig. S1†).

Each preparation, henceforth referred to as BSA–TCO_*x*_ (where *x* = TCO : BSA, rounded to the nearest integer), was purified and isolated in high recoveries (>85%), with no observable increase in the degree of aggregation compared to the starting material. Retention of TCO functionality was confirmed *via-* reaction of the TCO with a tetrazinecontaining Cy3 dye (Fig. S1†). Interestingly, we observed that pre-incubation of native BSA in an EDTA containing buffer (BBS–EDTA) was necessary to obtain efficient iEDDA reactivity. We hypothesize that commercial BSA formulations contain metal impurities (*e.g.*, copper), which could catalyse the isomerization of *trans*-cyclooctene into the inactive *cis*-cyclooctene, and thus decrease reactivity. Results supporting this hypothesis have been previously reported.^25,26^

### Preparation of BSA–AuNP nanoparticles

**Fig. 2:**
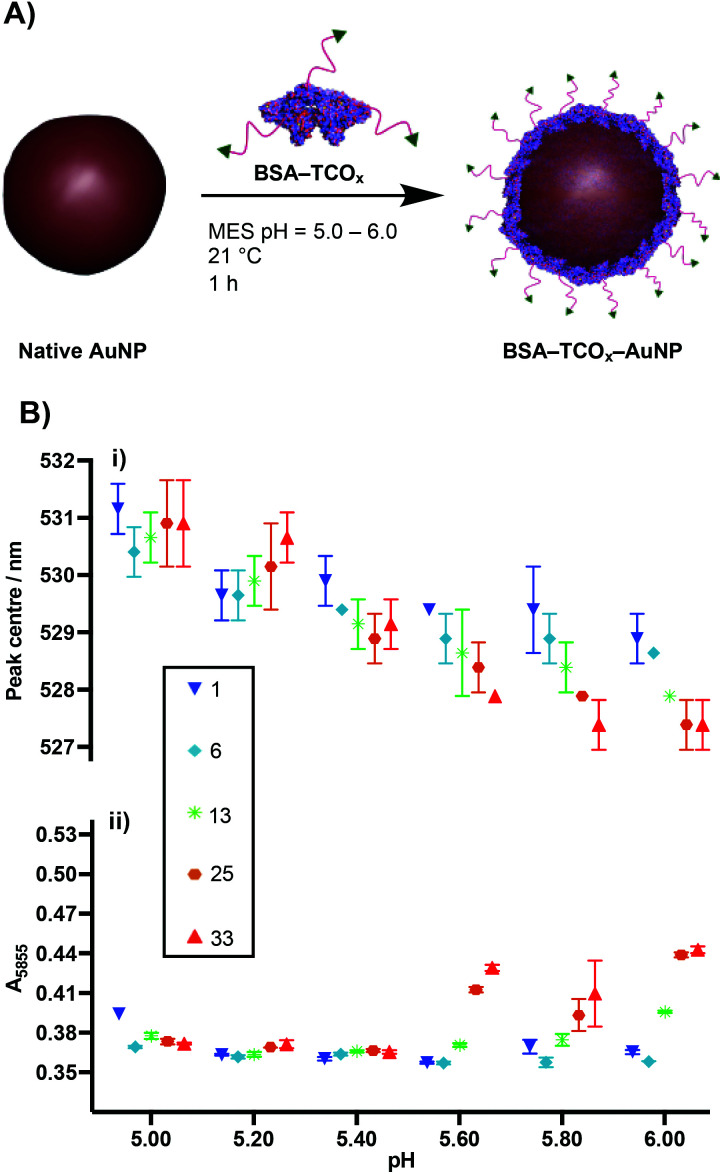
(A) Schematic representation of physisorption of BSA–TCO_*x*_ onto AuNPs. (B) UV-Vis data of physisorption of BSA–TCO_*x*_ to 40 nm citrate-capped AuNPs (*N* = 3, mean ± SD); (i) plasmon peak centre *vs*. pH, (ii) measure of flocculation (*A*_585_) *vs*. pH of BSA–TCO_*x*_–AuNP particles subjected to high salt concentration. The numbers in the legend (square box) represent the TCO : BSA ratio.

The physisorption of BSA onto AuNP surfaces has been well-studied, and multiple models exist describing the governing interactions. The prevailing theory posits that as the protein makes contact with the gold surface, it partially denatures, adopting a flatter conformation with a larger surface area.^27–29^ These changes facilitate a strong interaction between the protein and the gold surface, either *via* non-covalent electrostatic forces or a thiol–Au bond.^21^ These electrostatic forces are formed in part through the charged lysine residues. Thus, we decided to determine the effect that functionalising lysine residues has on the interaction of BSA and gold nanoparticles. We incubated the different formulations of BSA–TCO_*x*_ with 40 nm citrate-capped AuNPs at pH values ranging from 5.0–6.0, and observed changes in the surface plasmon of the gold nanoparticles using UV-Vis spectroscopy (Fig. 2i and Fig. S2†).

The formation of a protein corona on the surface of gold nanoparticles is known to cause a red-shift in the surface plasmon resonance, and thus acts as an indication of successful physisorption.^30^ Similarly, protein corona formation is known to impart resistance to flocculation in high salt content buffers. Thus, flocculation measurements (*A*_585_) act as a proxy measurement for protein corona formation (Fig. 2ii and Fig. S2†), with higher *A*_585_ values indicating increased flocculation.^31,32^ Since differences between certain BSA–TCO_*x*_–AuNP formulations prepared at pH = 5.20 and pH = 5.40 were difficult to ascertain *via* UV-Vis spectroscopy, we assessed these particle formulations using dynamic light scattering (DLS) (Fig. S3†). Once again, no significant differences were observed.

The data suggest that physisorption of BSA–TCO_*x*_ is more favourable at lower pH values and lower degrees of functionalisation, though at pH = 5.20 and 5.40 stable BSA–TCO_*x*_–AuNP complexes form for each of the BSA–TCO_*x*_ formulations.

At higher pH values BSA–TCO_*x*_ formulations with a high TCO : BSA ratio demonstrated a limited ability to adhere to the gold surface, as evidenced by a minimal shift in the plasmon peak and a high degree of flocculation in high salt buffers. This can easily be rationalised in the context of an electrostatic interaction model for the physisorption of BSA to gold nanoparticles. At lower pH and low degrees of functionalisation, more positively charged lysine residues are available to mediate a stronger interaction with the negatively charged citrate-capped AuNPs. At higher pH and high degrees of functionalisation the lysine residues are either negatively charged or capped with TCO, the overall positive charge of the protein is decreased, and affinity for the surface decreases. A plot of degree of functionalisation of *f*-BSA against calculated isoelectric point (Fig. S4†) matches the trend observed in Fig. 2B, particularly at high pH values, supporting this theory. Based on the collective data, we decided to move ahead with an optimised pH of 5.20, and a TCO : BSA ratio of ∼25 to produce BSA–TCO_25_–AuNPs.

**Fig. 3:**
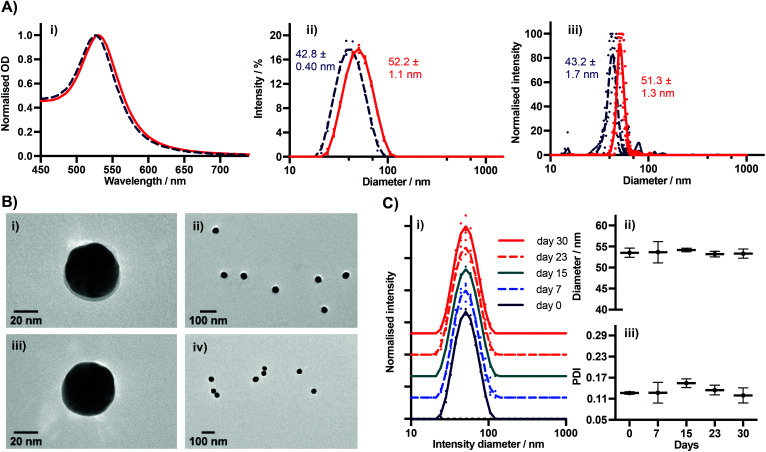
(A) (i) UV-Vis, (ii) DLS, and (iii) NTA spectra of BSA–TCO_25_–AuNPs prepared at pH = 5.20 (*N* = 3). Native 40 nm AuNPs included (dashed line) for comparison. For DLS and NTA, individual measurements are plotted, with a line representing the mean. (B) TEM of nanoparticles; (i and ii) BSA–TCO_25_–AuNPs. The BSA–TCO_25_ corona can be clearly seen in (i); (iii and iv) Native AuNPs showing no corona. (C) DLS data showing stability of BSA–TCO_25_–AuNPs over time (*N* = 3). (i) Normalised intensity spectra. Spectra have been artificially shifted on the *Y* axis for clarity. Individual measurements are plotted, with a line representing the mean. (ii) Intensity diameter *vs*. storage time, (iii) PDI *vs*. storage time.

**Fig. 4:**
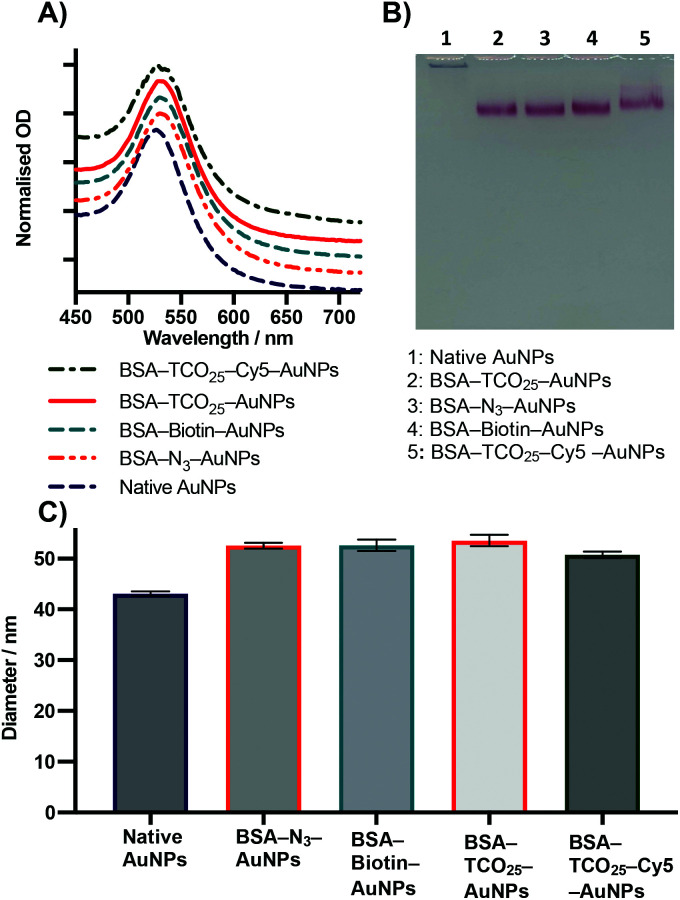
Characterisation of 40 nm AuNPs coated with BSA functionalised with various chemical moieties. (A) UV-Vis data. Lines are an average of 3 separate preparations. (B) Agarose gel electrophoresis. (C) DLS intensity diameter. Data plotted as mean ± SD (*N* = 3).

We characterised BSA–TCO_25_–AuNPs using UV-Vis spectroscopy, dynamic light scattering (DLS), nanoparticle tracking analysis (NTA), TEM (Fig. 3), and agarose gel electrophoresis (Fig. 4). The UV-Vis spectra show a clear red-shift in the plasmon position and no significant shouldering, evidencing both successful corona formation and a lack of aggregation. Particle diameters of 52.2 ± 1.1 nm and 51.3 ± 1.3 nm were obtained from DLS and NTA analysis, respectively, for BSA–TCO_25_–AuNPs. TEM images of the particles clearly show the presence of a protein corona that is not observed on the native control particles. Differences in the migration of native citrate-capped gold nanoparticles and BSA–TCO_25_–AuNPs also evidence a successful reaction (Fig. 4). We determined the long-term stability of BSA–TCO_25_–AuNPs by monitoring changes in the diameter and polydispersity of the particles over 30 days; no significant differences were recorded (Fig. 3C). Titration of BSA–TCO_25_ against citrate-capped AuNPs suggests that the interaction is comparable with similar protein–gold systems reported in the literature *e.g.*, glutathione S-tranferase (Fig. S5†).^18^

Collectively, these data are consistent with the formation of a monolayer corona of *f*-BSA on the gold nanoparticle surface. The diameter increase (*Δ*_D_) of 9.4 ± 1.5 nm and 8.1 ± 3.0 nm obtained from DLS and NTA, respectively, is in agreement with previous studies.^27^ Though slightly lower than the theoretical increase expected, it is known that conformational changes can occur upon interaction of BSA with gold surfaces.^27^ Though it is clear that over-functionalisation of BSA can decrease the protein's affinity for gold surfaces (Fig. 2), this data demonstrates that a strong interaction still occurs provided the degree of functionalisation is kept relatively low. The lack of any aggregation over a 30 day period suggests that *f*-BSA–AuNPs have good long-term storage potential; this is an important factor for many applications.

### Testing the scope

After optimization of the physisorption of functionalised BSA to gold nanoparticles using BSA–TCO_25_, we wanted to determine whether the method could be generalised to produce functionalised gold nanoparticles with different chemical functional groups. We prepared BSA–N_3_, BSA–biotin, and BSA–Cy5 (see Methods section for details) and subsequently incubated each of them with citrate-capped gold nanoparticles according to the optimised protocol (Fig. 4).

In each case, we observed the expected diameter and plasmon resonance shift, with no apparent aggregation. No significant difference in either diameter or polydispersity was observed between the different *f*-BSA–AuNP formulations, and their migratory behaviour on agarose gel was also similar. We suspect the slight difference in migration of BSA–TCO_25_–Cy5 (lane 5) results from the positive charge of Cy5 retarding the migration of the complex to the anode. These experiments demonstrate the generality of this approach for installing a variety of functional groups onto the surface of gold nanoparticles, using simple, cheap, and commercially available reagents. By simply employing a different NHS ester during the preparation of *f*-BSA the system can be rapidly adapted to the user's needs.

### Stability of BSA–AuNP nanoparticles

**Fig. 5:**
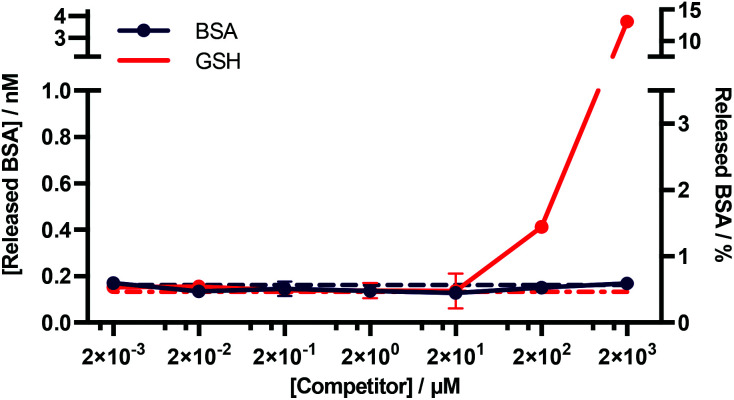
Quantitative ELISA measuring the levels of BSA–biotin released from BSA–biotin–AuNPs after titration with native BSA and GSH. Data plotted as mean ± SD (*N* = 3). The dotted lines indicate the background signal ([Competitor] = 0 mM). The percentage of BSA released (right *y*-axis) is based on a theoretical calculation.†

An important consideration for many diagnostic applications is the stability of *f*-BSA-functionalised gold nanoparticles in the presence of common plasma components. We were particularly interested in studying the interactions of the particles with albumin and blood thiols *e.g.* glutathione. Theoretically, both albumin and reduced glutathione (GSH) could compete with functionalised BSA for binding sites on the gold surface, and thus lead to desorption of the functionalised BSA. To test for this, we designed an experiment to determine the degree of desorption of *f*-BSA from AuNP surfaces in the presence of both albumin and reduced GSH. We incubated BSA–biotin–AuNPs with increasing concentrations of the competitors (0.002–2000 μM) for five hours, after which time the amount of BSA–biotin in solution was quantified (Fig. 5). BSA–biotin in solution was measured using a sandwich ELISA assay with streptavidin–HRP, and quantified against a standard curve of BSA–biotin.

The results of this assay suggest that no desorption of BSA–biotin occurs after incubation with native BSA across the entire concentration range, and approximately 0.4 and 4 nM of BSA–biotin was released from the particle surface after incubation with 200 and 2000 μM of reduced GSH, respectively, after five hours of incubation. This corresponds to approximately 1.5% and 15% of the total bound BSA–biotin. The concentration of albumin in plasma and serum is typically 525–750 μM.^33^ GSH concentrations are in the range of 1–25 μM, below the experimentally obtained threshold for desorption.^34–36^ Thus, these data demonstrate that *f*-BSA–AuNPs are stable at and above the albumin and GSH concentrations commonly present in blood plasma, over timescales that far exceed those of a typical *in vitro* diagnostic assay.

### BSA–AuNP–IgG conjugation

The optical and plasmonic properties of gold nanoparticles provide a simple and easily-interpretable readout for both *in vitro* and *in vivo* diagnostics, facilitating the generation of diagnostic data using common lab equipment and minimal expertise. For many common diagnostic tests it is necessary to impart disease-recognition elements to the particles, generally through the incorporation of target-specific ligands such as antibodies.^4,37^

To this end, we wanted to determine whether it was possible to attach antibodies to functionalised BSA–AuNPs, and whether these complexes would retain their ability to specifically target disease.

We chose to use the antibody Ontruzant (ONT) as a model IgG. Ontruzant is a monoclonal humanised IgG against the breast cancer biomarker HER2, and is currently marketed as a biosimilar for the popular therapeutic Herceptin™. As a validated and approved antibody for both diagnostic and therapeutic applications, Ontruzant™ is an ideal model IgG to validate this system.

**Fig. 6:**
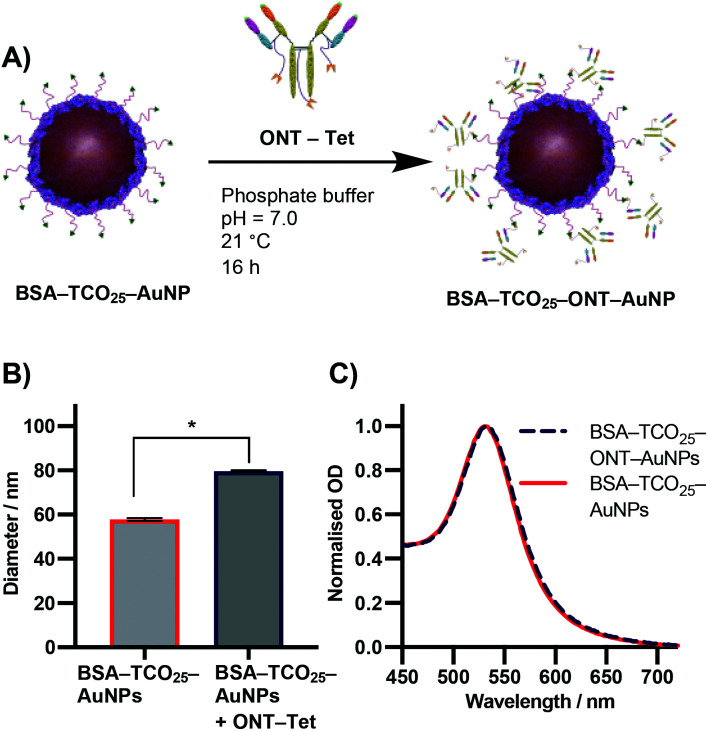
(A) Schematic representation of the iEDDA reaction between BSA–TCO_25_–AuNPs and ONT–Tet. (B) DLS data showing the diameter of BSA–TCO_25_–AuNPs after addition of ONT–Tet. Data plotted as mean ± SD (*N* = 3). Statistical significance * (*P* = 0.0001) was determined using an unpaired *t*-test. (C) UV-Vis spectra comparing BSA–TCO_25_–AuNPs and BSA–TCO_25_–ONT AuNPs.

Due to the previously detailed benefits, iEDDA was chosen as the ligation chemistry of choice for preparation the AuNP–IgG conjugates. BSA–TCO_25_–AuNPs were prepared according to the optimised protocol, and the corresponding tetrazine click partner was installed onto ONT using disulfide-selective pyridazinedione chemistry. A methyltetrazine-bearing dibromopyridazinedione was synthesized according to a reported procedure,^38^ and subsequently conjugated to ONT using established protocols (Fig. S6–S17†).^39^ The optimal pH, concentration and time for conjugating tetrazine-modified Ontruzant (ONT–Tet) to BSA–TCO_25_–AuNPs were determined by incubating the two components together and then measuring the binding to their target (Fig. S18†). To provide a reference for the performance of the iEDDA chemistry, simple physisorption of ONT onto citrate-capped 40 nm AuNPs was performed in parallel. Physisorption of IgGs onto AuNPs is the standard method for AuNP–IgG conjugation, thus provides a suitable reference. Incubation of ONT–Tet and BSA–TCO_25_–AuNPs under optimal conditions resulted in a statistically significant increase of 21.7 ± 0.88 nm in particle diameter, with no associated aggregation (Fig. 6B). In contrast, the control reaction of native ONT and BSA–TCO_25_–AuNPs showed no significant difference in diameter. We quantified the number of antibodies installed on the particles using a 3-(4-carboxybenzoyl)quinoline-2-carboxalde-hyde (CBQCA) assay, obtaining a value of 43.4 ± 7.8 IgG per particle. For comparison, we obtained a value of 40.7 ± 4.7 IgG per particle from the physisorption of ONT to native citrate-capped AuNPs (Fig. S19†). The total particle recovery was consistently greater than 90%, even after multiple washing steps.

These experiments suggest that the conjugation of BSA–TCO_25_–AuNPs and ONT–Tet occurs *via* the suspected iEDDA mechanism, and preclude the presence of any unwanted non-specific reactions. Additionally, under optimal conditions the conjugation of ONT–Tet to BSA–TCO_25_–AuNPs leads to the same IgG density as physisorption of ONT (43.4 ± 7.8 *vs*. 40.7 ± 4.7 IgG per particle, Fig. S19†). To the best of our knowledge, this is the first reported case of iEDDA chemistry being used to conjugate an IgG to gold nanoparticles; thus, this represents an important step forward in the field of nanoparticle–protein conjugation. Though previous catalyst-free click chemistries, *e.g.*, strain-promoted alkyne–azide click (SPAAC), have routinely been employed for this purpose, the poor reaction kinetics often necessitate high concentrations or large excesses of the antibody to achieve a satisfactory degree of conjugation. By enabling the use of iEDDA chemistry, this approach could offer a much more efficient means for preparing protein-conjugated gold nanoparticles; this work is currently being explored further in our lab.

### Antigen targeting of BSA–AuNP–IgG complexes

**Fig. 7:**
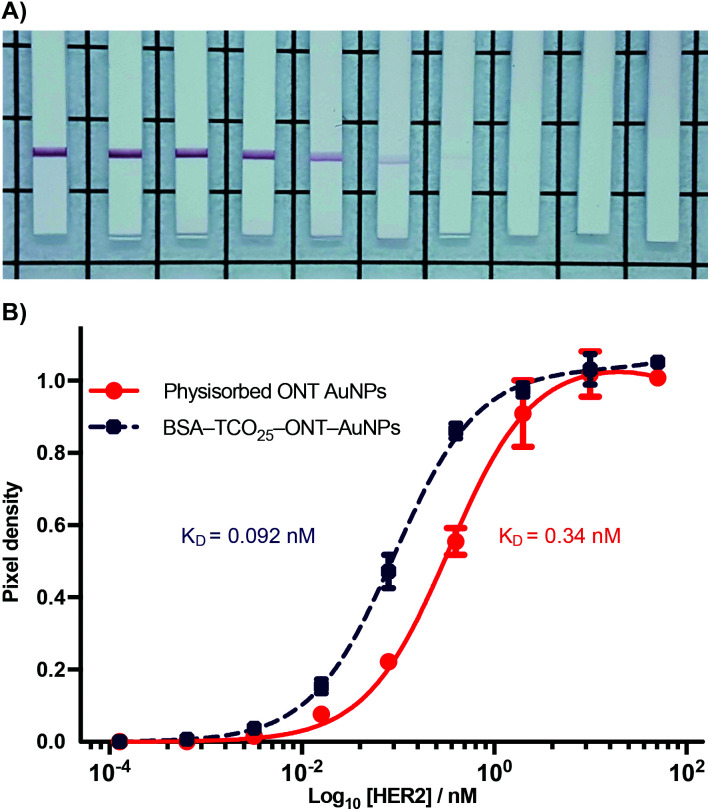
Sandwich LFIA of BSA–TCO_25_–ONT–AuNPs and physisorbed ONT-AuNPs binding to biotinylated HER2, captured by a polystreptavidin coated test line. (A) Photos of nitrocellulose test strips showing a positive test line from binding of BSA–TCO_25_–ONT decreasing HER2 concentration left to right, 10–0.000128 nM + 0. See or raw images of all test strips. (B) log_10_[HER2] *vs*. signal, calculated from the pixel density at the test line. Lines were fit using non-linear regression, with *K*_D_ calculated from a binding saturation isotherm. Data plotted as mean ± SD (*N* = 3).

During the optimization of the conjugation of ONT–Tet and BSA–TCO_25_–AuNPs, we observed that the resulting particles had an apparently higher binding to HER2 relative to particles prepared *via* physisorption of unmodified ONT to citrate-capped gold nanoparticles (Fig. S18†). To explore this further, we designed a model paper-based lateral flow assay. To enable detection of HER2 in a sandwich format we used polystreptavidin as the capture ligand, and employed a biotinylated HER2 to complete the sandwich complex. We titrated solutions of biotinylated HER2 (1.28 × 10^−4^–10.0 nM) against preparations of BSA–TCO_25_–ONT–AuNPs and ONT–AuNPs prepared *via* physisorption, and subsequently flowed the solutions up nitrocellulose strips containing a polystreptavidin test line. Binding of the particles at the test line was determined using densitometry (Fig. 7).

A plot of HER2 concentration against test line intensity shows a clear difference in target binding between the two approaches. BSA–TCO_25_–ONT–AuNPs clearly bind more strongly, with a *K*_D_ of 0.092 ± 0.0043 nM compared to 0.34 ± 0.023 nM for physisorbed ONT–AuNPs. No statistical significance in the number of antibodies per particle between the two preparations was observed (Fig. S19†), so we can infer that the observed difference in affinity is not attributable to differences in ligand density. Thus, it stands to reason that we can attribute the observed differences in affinity to either a greater availability of antibody paratopes (*i.e.*, a higher fraction of active antibodies), or that individual paratopes are better able to bind to the target; of course, it could be a combination of both factors. It is feasible that greater paratope availability in BSA–TCO_25_–ONT–AuNPs could result from a more favourable orientation of the ONT ligands afforded by the site-directed pyridazinedione-mediated conjugation; this has certainly been demonstrated with other nanoparticle systems.^40–42^ However, due to the presence of multiple disulfide bonds within the IgG scaffold, the pyridazinedione chemistry still presents four sites for conjugation (albeit four sites distal to the antigen binding paratope), so there is still some degree of heterogeneity in the attachment.^43^ This could minimise any gains achieved from favourable orientation. Perhaps a more likely explanation for a higher number of active antibodies is that preparation of the particles *via* the covalent *f*-BSA approach leads to less IgG denaturation compared to particles prepared *via* physisorption. In the case of BSA–TCO_25_–ONT–AuNPs, the layer of BSA between the gold surface and the IgG could protect against denaturation, leading to a higher degree of active ligands. An alternative argument is that the greater affinity is a consequence of increased ligand flexibility, resulting from the layer of BSA and PEG linker. The added flexibility between the AuNP surface and the recognition element could decrease the steric barriers to binding by allowing the immunocomplex to form at a greater distance from the particle surface. Further experiments are underway to elucidate the mechanisms that lead to improved antigen binding.

## Conclusion

To conclude, we have described a simple, cheap, and effective method for introducing chemical functionality to gold nanoparticles whilst simultaneously providing protection against particle fouling. By exploiting the abundance of lysine residues on BSA we were able to generate bioconjugates (*f*-BSA) with a defined number of functional groups, and subsequently exploit the strong interaction of BSA with gold to deposit them onto the surface of AuNPs. This method was shown to be generalisable, enabling the introduction of *trans*-cyclooctene, azide, biotin, and the fluorescent dye Cy5 to the nanoparticle surface. The resulting nanoformulations displayed excellent stability within complex media, and no measurable aggregation during long-term storage was observed. The utility of *f*-BSA–AuNPs was demonstrated through the subsequent attachment of Ontruzant, a clinically relevant antibody against the breast cancer biomarker HER2. The particles generated *via f*-BSA–AuNPs displayed significantly increased binding to the target when compared to particles generated using the traditional approach of physisorption; we are currently exploring this particular feature of *f*-BSA–AuNPs in more depth. When considering the ubiquitous use of gold nanoparticles within lateral flow immunoassays, this data suggests that this novel approach could hold significant value in the development of paper-based diagnostics. Moreover, the simplicity and generalisability of this approach offers an easy route towards functionalising gold nanoparticles, a fact we expect will engender broad adoption of the method for a variety of applications.

## Materials & methods

### General

All buffers were prepared using Milli Q d.d. H_2_O, and filtered through a 20 μm filter before use. Buffer pH was adjusted using 2 M HCl and 2 M NaOH, and tested each time before use. Gold nanoparticle solutions were handled and stored in Protein LoBind® sample tubes (Eppendorf) or glass sample vials, when large volume storage was required. Centrifugal ultrafiltration was performed using Amicon®-Ultra devices (Merck) of an appropriate volume. The synthesis and characterization of dibromopyridazinedione-methyltetrazine is detailed in the ESI.†

### Buffers

The following buffers were used routinely: MES buffer (pH = 5.0–6.0, 20 mM 2-ethanesulfonic acid); BBS (pH = 8.0–10, 5.0–50 mM borate, 25 mM NaCl); BBS–EDTA (pH = 8.0, 50 mM borate, 25 mM NaCl, 2.0 mM ethylenediaminetetraacetic acid); PBST (PBS + 0.1% tween 20); carbonate buffer (pH = 9.8, 20–100 mM carbonate); phosphate buffer (pH = 7.0, 20 mM phosphate); phosphate storage buffer (pH = 7.0, 20 mM phosphate, 0.05% tween 20); carboante storage buffer (pH = 9.8, 20 mM, 0.05% tween 20); blocking buffer (pH = 9.8, 100 mM carbonate, 2% beta-casein).

### Statistics and curve fitting

Statistical significance (*P* = 0.0001) between the diameters of nanoparticle solutions (Fig. 6B) was determined using an unpaired *t*-test. Normality was determined using the Shapiro–Wilk test. The equilibrium binding constant (*K*_D_) between BSA–TCO_25_ and citrate-capped 40 nm gold (Fig. S5†) was obtained by fitting the data to the following equation:
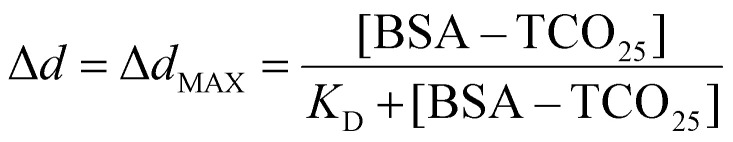


To enable quantitative and qualitative comparison, UV-Vis spectra for AuNPs were normalized to the highest point of the plasmon peak and fitted to a cubic spline. Peak centre and *A*_585_ were read directly from the spline.

### Dynamic light scattering

DLS measurements were performed on a Zetasizer Nano ZS (Malvern) equipped with a 633 nm laser. Measurement parameters were optimised by the Zetasizer Nano software v3.30, and samples were equilibrated to 21 °C before measurements. Equilibrium binding data (Fig. S5†) was obtained by incubating separate particle solutions with the described concentration of BSA–TCO_25_ for one hour at 21 °C and then measuring the particle diameter using DLS. Values for the size *vs.* intensity, intensity diameter, *Z*-average diameter, and polydispersity index were calculated by the software and exported without further manipulation. Changes in diameter (Δ*d*) were calculated as the difference in intensity diameter between the product and starting material.

### Nanoparticle tracking analysis

Nanoparticle tracking analysis (NTA) was performed on a Nanosight NS3000 (Malvern) equipped with a 532 nm laser and sCMOS camera. Particles were diluted into d.d. H_2_O (10^7^–10^8^ particles per mL). All particle solutions were equilibrated to 21 °C before measurements. 60 seconds videos were recorded per sample, using NTA software V3.0 with a camera level of 8 and a detection threshold of 21. Mean values and errors were calculated by the software and exported without further manipulation.

### TEM

AuNP samples for TEM were prepared by suspending the AuNPs in ultra-pure distilled water, and diluting down to a concentration of ∼5 pM. Then 1 μL of sample solution was added to a TEM copper grid (CF-400-Cu, Electron Microscopy Sciences) which was then dried in air for 30 min. Nanoparticle TEM samples were imaged on a JEOL 2100Plus Transmission Electron Microscope at 200 kV with a beam current of 101 μA. TEM micrographs were captured with the Gatan Orius SC1000 camera at magnifications of 25 000× and 60 000×.

### MALDI

A solution of BSA or BSA–TCO_*x*_ in water was diluted with a solution of sinapinic acid (saturated in 1 : 1 H_2_O/MeCN with 0.01% formic acid) to concentration of approximately 2 μM. A MALDI plate was prepared by spotting 1 μL of sinapinic acid solution on each well that was to be used. After air-drying, 1 μL of diluted BSA or BSA–TCO_*x*_ sample was added to the prepared wells. After air-drying, the plate was inserted into a Waters Micromass MALDI micro MX Mass Spectrometer. Spectra were acquired in linear positive mode, with laser power: 381%, pulse voltage: 2000 V, detector voltage: 2500 V, suppression: 10 000 Da, sample period: 1 ns, sensitivity: 100 mV and TLF delay: 1425 ns. Data was analysed by MassLynx V4.1. Background was subtracted (polynomial order: 1, below curve: 40%, tolerance: 0.01) and spectra were smoothed (smooth window: ±10, number of smooths: 100, smoothing method: mean).

### UV-Vis spectroscopy

UV-Vis were obtained on a Nanodrop 2000c (Thermo Scientific™) running in cuvette mode, scanning 200–840 nm in 1 nm steps. Baseline correction was achieved by running a blank containing only the sample buffer, and automatically subtracted from the data by the software. Samples were measured in microcuvettes with a path length of 1 cm. For high-throughput parameter exploration spectra were obtained in clear plastic 96 well plates using a SpectraMax M5 (Molecular Devices), scanning 500–840 nm in 5 nm steps. Baseline correction was achieved by running a blank containing only the sample buffer and subtracting during data analysis.

### SDS-PAGE

SDS-PAGE was performed on non-reducing gels comprising 10% bis-acrylamide (40% acrylamide/bis solution, Bio-Rad), 0.1% sodium dodecyl sulfate, 0.1% ammonium persulfate and 0.01% tetramethylethylenediamine in 1.5 mM Tris·HCl pH = 8.8. A stacking gel comprising 6% bis-acrylamide, 0.1% sodium dodecyl sulfate, 0.1% ammonium persulfate, and 0.01% tetramethylethylenediamine in 0.5 mM Tris·HCl pH = 6.8 was used. Samples (1 mg mL^−1^ in d.d. H_2_O) were pre-mixed 4 : 1 with 4× Laemmli sample buffer (Bio-Rad), incubated at 75 °C for 5 minutes, and then centrifuged (10 000 r.c.f, 2 minutes). A pre-stained protein ladder (PageRuler™ Protein Plus, Thermo Scientific™) was included. The samples (4 μL) were loaded onto the stacking gel, and electrophoresis was performed at constant amperage until complete. The gel was stained overnight (QC colloidal Coomassie stain, Bio-Rad), destained using 20% MeOH in d.d. H_2_O, and then imaged.

### Agarose gel electrophoresis

Agarose gel electrophoresis was performed on gels comprising 1.5% agarose (UltraPure™ Agarose, Invitrogen™) in Tris-borate–EDTA (TBE) buffer, pH = 8.3. The samples (20 μL, OD = 10, phosphate buffer pH = 7.0 0.05% tween 20) were loaded at the anode, and electrophoresis was performed in TBE buffer at 80 V for 2 hours. The gel was imaged without further manipulation.

### Lateral flow sandwich assays

Solutions of BSA–TCO_25_–ONT–AuNPs and ONT–AuNPs (OD = 1, 20.0 μL, carbonate storage buffer) were added to a 96 well plate and equilibrated to 21 °C. A dilution series of biotinylated HER2 (1.28 × 10^−4^–10.0 nM, SinoBiological) was prepared in foetal bovine serum (0.1% tween 20), and added to the nanoparticle solutions. After incubation at 21 °C for 5 minutes, nitrocellulose strips (NC-95 membrane, grade 270 absorbent pad, 3 mm width, 33 mm length, Mologic Ltd) containing a printed polystreptavidin test line were submerged into the solutions. The solution was allowed to wick up the strips until completely consumed (10 minutes). The strips were air-dried for 30 minutes, placed onto a backing grid, and photographed under white light using a Canon PowerShot SX720 HS. Test line densitometry was achieved using Image J. Briefly, the raw images were imported into Image J and converted to grey-scale. A region of interest (ROI) was drawn around the test line, and the pixel density counted using the software. To control for lighting differences across the image, an identical ROI was drawn around the printed grid line directly below each strip, and the pixel density from the test lines normalized to these values.

### Preparation of copper-free BSA

BSA (5.00 g, Sigma Aldrich) was dissolved in BBS pH = 8.00 (2 mM EDTA, 100 mL) and incubated at 4 °C for 16 hours. The protein was subsequently dialysed (10 000 Da MWCO, 4 × 15.0 mL) into BBS pH = 8.0 (2 mM EDTA), and left for a further 5 hours at 21 °C. The protein was once again dialysed (10 000 Da MWCO, 4 × 15.0 mL) into BBS pH = 8.0 without EDTA, flash frozen, and stored at −20 °C.

### Functionalisation of BSA

This protocol describes the preparation of BSA–TCO_25_. Modification of BSA with biotin (NHS–PEG_4_–biotin, Jena Biosciences) and azide (NHS–PEG_4_–azide, Jena Biosciences) was achieved using the same protocol.

To a solution of copper-free BSA (2.00 mL, 72.2 μM, BBS pH = 8.00), *trans*-cyclooctene–PEG_4_–*N*-hydroxysuccinimide (144 μL, 50 equiv., 50 mM in DMSO, Jena Biosciences) was added and the solution incubated at 21 °C for 7 hours. The protein was subsequently purified *via* ultrafiltration (10 000 Da MWCO, 6 × 2.00 mL) into d.d. H_2_O, flash frozen, and stored at −20 °C. Protein concentration was determined using UV-Vis (*ε*_280_ = 43 824 M^−1^ cm^−1^).

### Preparation of BSA–TCO–Cy3 and BSA–TCO–Cy5

To a solution of BSA–TCO_25_ (50.0 μM, BBS pH = 8.00), 6-methyltetrazine-Sulfo-Cy3/5 (25 eq., 50 mM in DMSO, Jena Biosciences) was added and the solution incubated at 21 °C for 2 hours. The protein was subsequently purified *via* ultrafiltration (10 000 Da MWCO, 6 × 2.00 mL) into d.d. H_2_O, flash frozen, and stored at −20 °C.

### Preparation of functionalised BSA–AuNPs

This protocol describes the preparation of BSA–TCO_25_–AuNPs. BSA–N_3_–AuNPs, BSA–biotin–AuNPs, BSA–TCO_25_–Cy5–AuNPs were prepared using the same protocol with the appropriate starting material. Details of the pH optimization can be found in the ESI (Fig. S2†).

To a solution of BSA–TCO_25_ (5.60 mL, 3.75 μM, MES pH = 5.20) in a 14 mL glass sample vial, citrate-capped 40 nm AuNPs (1.40 mL, OD = 5.00, BBI Solutions) were added and the solution incubated at 21 °C for 2 hours. Blocking buffer (0.700 mL) was added and the particles incubated at 21 °C for 30 minutes. The particle solution was subsequently separated into 8 Protein LoBind® Eppendorf tubes and centrifuged (5000 r.c.f., 10 minutes) to pellet the particles, which were subsequently washed (6 × 1.00 mL phosphate storage buffer). After the final wash the particles were resuspended into phosphate storage buffer (2.00 mL), briefly sonicated, and stored at 4 °C.

### Preparation of BSA–TCO_25_–ONT–AuNPs

To a solution of BSA–TCO_25_–AuNPs (135 μL, OD = 1.1 in phosphate storage buffer), ONT–Tet (15.0 μL, 400 nM in d.d H_2_O) was added and the solution left to incubate at 21 °C for 16 hours. The particle solution was subsequently centrifuged (5000 r.c.f., 10 minutes) to pellet the particles, and then washed (6 × 1.00 mL carbonate storage buffer). After the final wash the particles were resuspended into carbonate storage buffer, briefly sonicated, and stored at 4 °C.

### Preparation of ONT–AuNPs (physisorption)

To a solution of citrate-capped 40 nm AuNPs (100 μL, OD = 5.0, BBI International), ONT (400 μL, 50 nM in 20 mM carbonate buffer, pH = 9.80) was added and the solution left to incubate at 21 °C for 4 hours. Blocking buffer (50 μL) was added and the solution incubated at 21 °C for 30 minutes. The solution was subsequently centrifuged (5000 r.c.f., 10 minutes) to pellet the particles, which were then washed (6 × 1.00 mL carbonate storage buffer). After the final wash the particles were resuspended into carbonate storage buffer, briefly sonicated, and stored at 4 °C.

### Nanoparticle stability test

The stability of functionalised BSA–AuNPs was determined using an ELISA to determine the concentration of desorbed BSA–biotin in solution. The protocol is detailed in the ESI.†

## Data availability

Raw data are available at DOI: 10.5281/zenodo.5012729.

## Author contributions

DAR was responsible for the majority of the planning, organisation, and execution of the work, including the preparation and characterisation of modified proteins and nanoparticles, and optimisation of the lateral flow assay. MRT contributed to the planning of the work, as well as the optimisation of multiple key assays. PAS was responsible for MALDI optimisation and obtaining mass spectra of functionalised BSA. YC performed TEM characterisation of nanoparticle formulations. JF was responsible for protein modification and MALDI mass spectrometry. JCFN aided in the preparation of BSA-N_3_ and BSA-Biotin, and the resulting *f*-BSA–AuNP nanoparticles. VC provided guidance on protein modification and supervised PAS and JCFN. MMS helped with the planning of the work, provided guidance on nanomaterial characterisation, and supervised DR, MRT, YC, and JF.

## Conflicts of interest

There are no conflicts to declare.

## Supplementary Material

NR-013-D1NR02584H-s001
