# Supplementary material for: Employing defined bioconjugates to generate chemically functionalised gold nanoparticles for in vitro diagnostic applications
Source: Nanoscale. 2021 Jun 22;13(27):11921–31. doi: 10.1039/d1nr02584h (PMC8280965; doi:10.1039/d1nr02584h)
Supplement: NR-013-D1NR02584H-s001 [file NR-013-D1NR02584H-s001.pdf]

Electronic Supplementary Material (ESI) for Nanoscale.
This journal is © The Royal Society of Chemistry 2021

Supplementary Information

Employing defined bioconjugates to generate chemically functionalized gold nanoparticles

Daniel A. Richards, Michael R. Thomas, Peter A. Szijj, James Foote, Yiyun Chen, João C. F. Nogueira, Vijay Chudasama, and Molly M. Stevens*

Contents

General **2**
Synthesis 2
BSA–TCO$_X$ characterisation **3**
Optimisation of BSA–TCO$_X$–AuNP preparation **4**
Determination of isoelectric point of BSA **5**
***f*-BSA-AuNP equilibrium binding study** **6**
Organic synthesis **7**
Synthesis of methyltetrazine-OEG-amine 7
Synthesis of methyltetrazine-diBrPD 9
NMR spectra of chemical compounds **11**
Antibody modification **20**
pH optimisation **21**
BSA–TCO$_{26}$–ONT–AuNP pH optimisation 21
ONT–AuNP pH optimisation 21
Concentration optimisation **21**
BSA–TCO$_{26}$–ONT–AuNP concentration optimisation 21
ONT–AuNP concentration optimisation 21
Time optimisation **21**
BSA–TCO$_{26}$–ONT–AuNP time optimisation 21
ONT–AuNP time optimisation 21
Antibody per particle quantification **23**
Nanoparticle stability test **23**
Lateral flow strips **24**
References **24**

General

Synthesis

All reagents and starting materials were obtained from chemical suppliers, unless specifically stated otherwise, and were used as received. Reactions were monitored by thin layer chromatography using pre-coated SIL G/UV 254 plates purchased from VWR. Flash chromatography was carried out manually using Kieselgel 60 M 0.04/0.063 mm silica gel or automatically using a BioTage Isolera with KP-Snap or KP-Sil columns. NMR spectra were recorded using a Bruker AC300, AC500 or AC600 spectrometer (300 MHz, 500 MHz and 600 MHz respectively). Chemical shifts (δ) are given in ppm units relative to the solvent reference and coupling constants (*J*) are measured in Hertz. Proton (^{1}H) NMR multiplicities are shown as s (singlet), d (doublet), t (triplet), q (quartet), m (multiplet), dd (double doublet), dt (double triplet), etc. HMBC, HSQC and DEPT were employed to aid with accurate assignments. Infrared spectra were recorded on a Perkin Elmer Spectrum 100 FTIR spectrometer (ATR mode). Mass spectra of all synthetic organic molecules were obtained from the UCL mass spectroscopy service on a Thermo Finnigan MAT900Xp (EI and CI) mass spectrometer.

BSA–TCO$_X$ characterisation

TCO:BSA ratio was calculated according to the following equation, where BSA = 1:

$$TCO{:}BSA = \frac{Peakcenter_M - \mathrm{Peakcenter}_N}{399.5}$$

Peakcenter$_M$ represents the MALDI peak center of the modified BSA, and Peakcenter$_N$ represents the MALDI peak center of native BSA. The value of 399.5 represents the molecular weight (in g/mol) of the TCO moiety when attached to BSA.

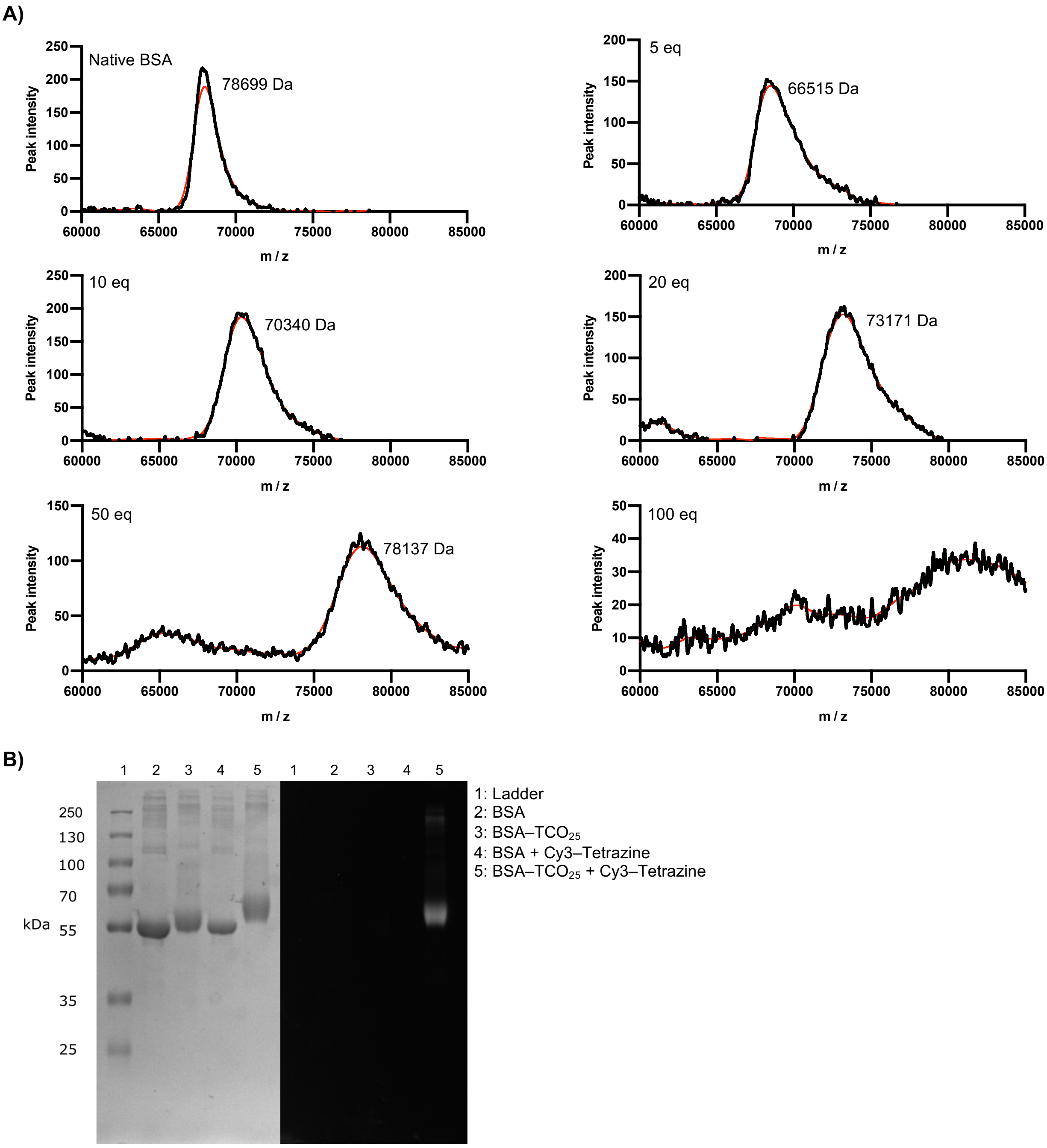

Fig. S1 A) MALDI spectra for BSA–TCO$_x$ constructs; red line represents a smoothed LOWESS curve with 20 points in the smoothing window. B) SDS-PAGE for the preparation of BSA–TCO$_{25}$. Click reaction with Cy3-tetrazine demonstrates the functionality of *trans-cyclooctene* on BSA through successful iEDDA click reaction.

Optimisation of BSA–TCO$_X$–AuNP preparation

Solutions of BSA–TCO$_X$ (x = 1, 6, 13, 25, 33, 60 µL, 5.0 µM in d.d. H_2O) were added in triplicate to a 96 well plate. To each set of triplicates MES buffer (20 µL, 100mM, pH = 5.0 – 6.0) was added, followed by 40 nm citrate-capped AuNPs (20 µL, OD = 5, BBI Solutions). The solutions were incubated at 21 °C for 2 hours, before the UV-Vis spectra were obtained on a plate reader. Subsequently, 10X PBS (40 µL) was added to each well to induce aggregation, and the solutions incubated at 21 °C for 2.5 hours before UV-Vis spectra were obtained.

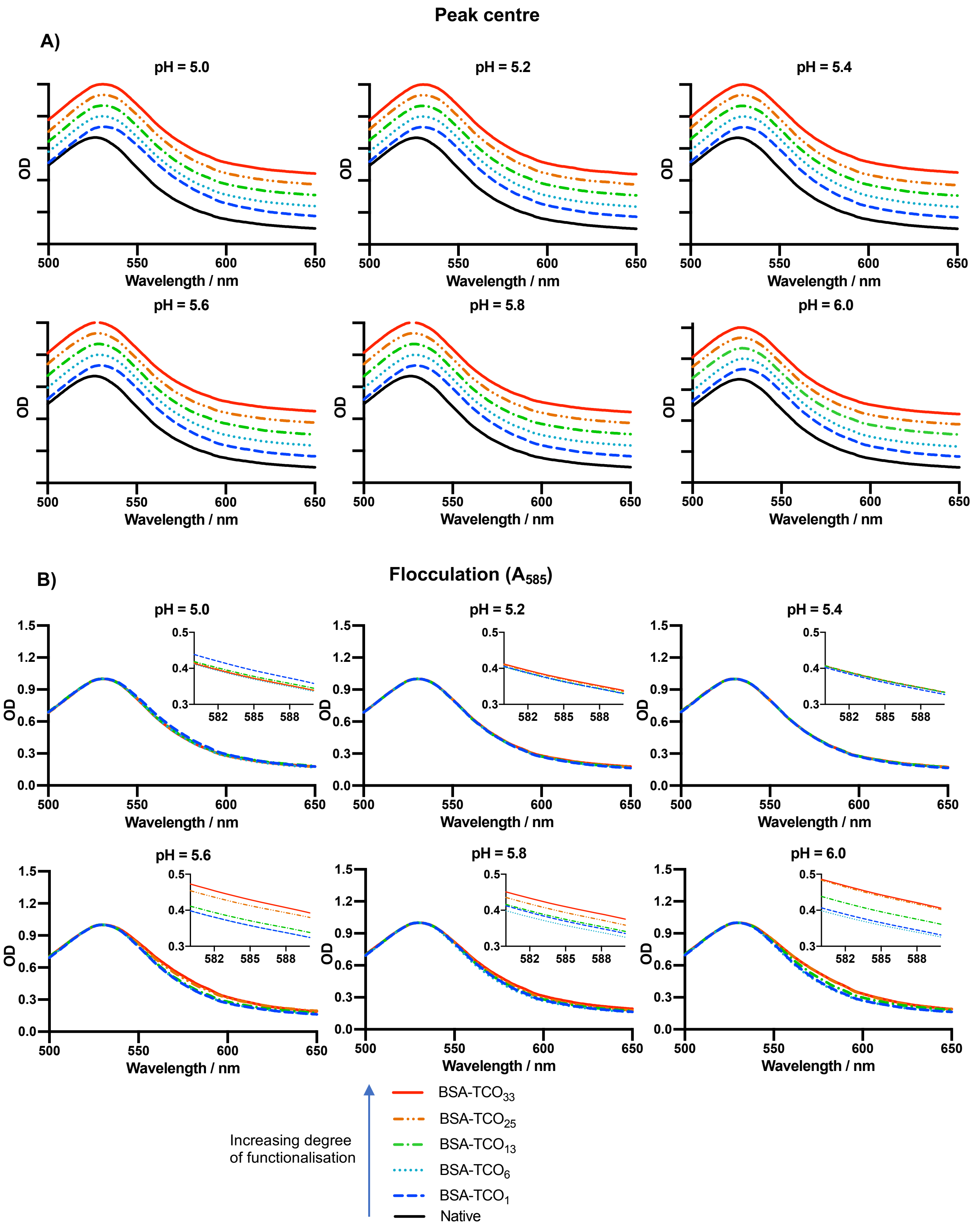

Fig. S2 UV-Vis data for the physisorption of BSA–TCO$_x$ onto citrate capped 40 nm AuNPs. Lines shown are a fitted cubic spline to the average (N = 3), normalised to the peak maximum. The native spectra is of citrate capped 40 nm AuNPs in d.d. H_2O. (A) Normalised spectra obtained during the physisorption at different pH values. Spectra are artificially shifted +0.1 OD (y axis) values relative to the native spectra for clarity. No significant aggregation is observed in any of the spectra. (B) Normalised spectra obtained after addition of 10X PBS. Insert shows the region of interest (585 nm) and the corresponding normalised OD values. Aggregation can be observed as both pH and degree of functionalisation increase. Spectra for native citrate-capped AuNPs are not included in the aggregation study as total aggregation and precipitation was observed.

Table S1: Tabulated data obtained from the UV-Vis data for the physisorption of BSA–TCO_x onto citrate capped 40 nm AuNPs (Fig. S2). 1: A_{585} values are from curves normalised to the peak maximum, and were obtained after addition of 10X PBS. 2: For Native AuNPs spectra were obtained in d.d. H_2O.

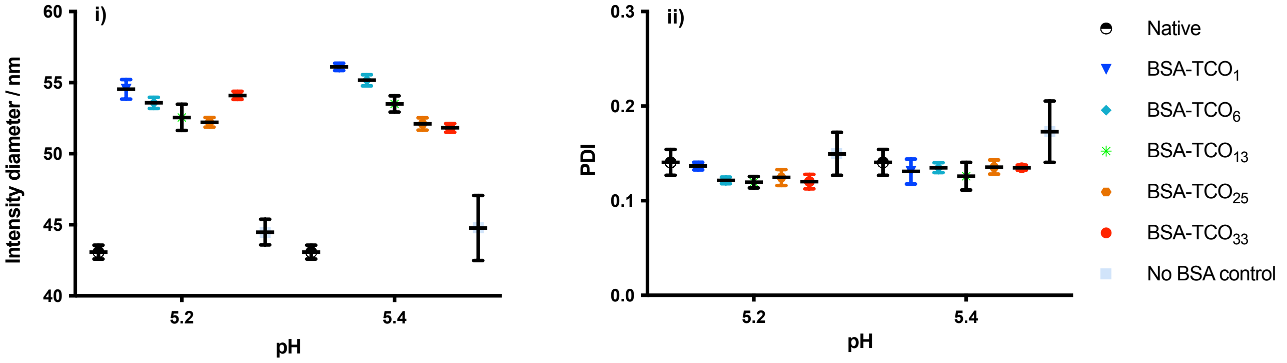

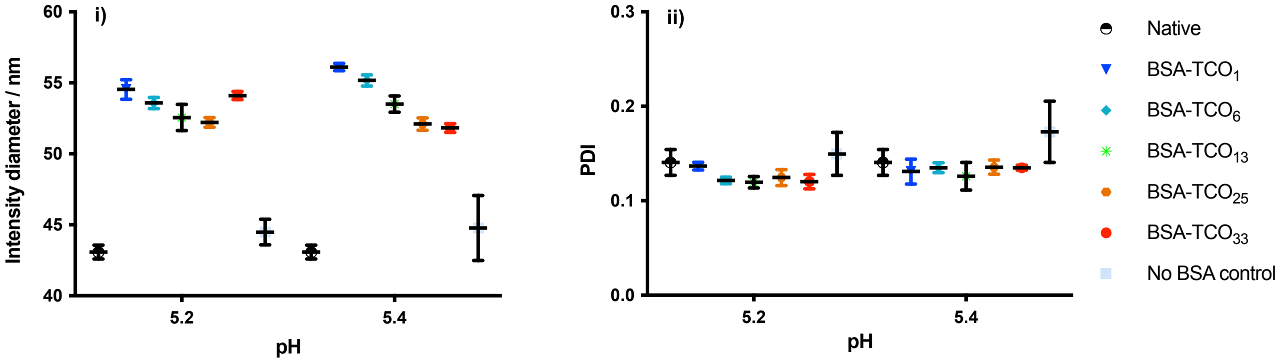

Fig. S3 Aggregated size intensity data obtained from DLS for the physisorption of BSA–TCO_x, functionalised to varying degrees, onto citrate capped 40 nm AuNPs in pH = 5.2 and pH = 5.4 MES buffer (i) Intensity diameter, (ii) polydispersity index (PDI). Data plotted as mean ± SD (N = 3, n =12).

Determination of isoelectric point of BSA–TCO_X

The amino acid sequence for BSA was obtained from UniProt (A0A140T897_BOVIN), and the isoelectric point determined using the ExPasy Compute pI/Mw tool (web.expasy.org/compute_pi). To simulate capping of the lysine residues with NHS-PEG_4-TCO, random lysine residues were replaced with glycine residues (1 – 40 replacements), and the isoelectric point calculated again. The data was plotted as degree of functionalization *vs* isoelectric point.

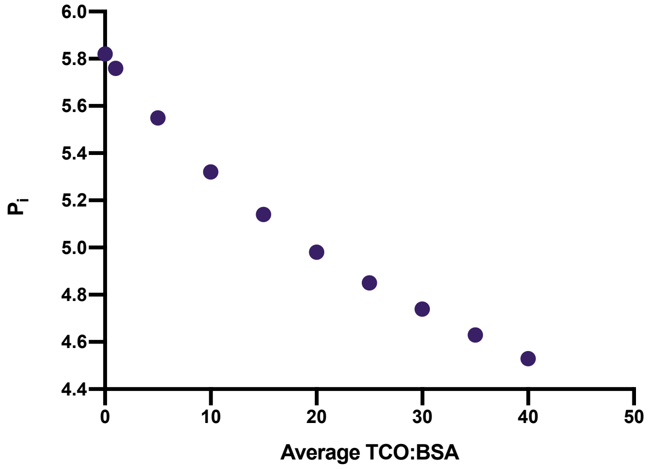

Fig. S4 Average TCO:BSA vs calculated isoelectric point.

f-BSA-AuNP equilibrium binding study

Adsorption of *f*-BSA to 40 nm citrate-capped AuNPs was performed according to the procedure described in the manuscript, though the concentrations of *f*-BSA were varied accordingly. An equilibrium binding constant (K_D) of 16.36 ± 0.12 nM was obtained after fitting the data. This is comparable with similar protein–gold systems reported in the literature e.g., Glutathione S-Tranferase (ca. 50 – 80 nM).[1]

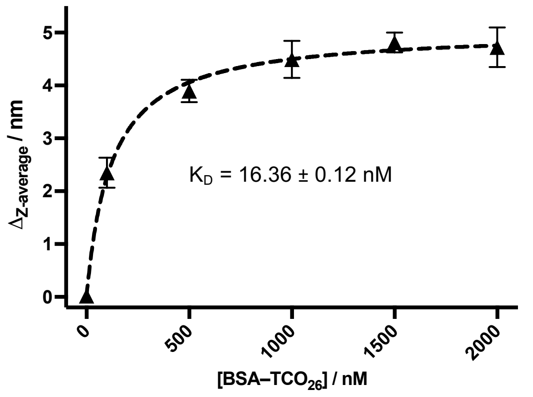

$$(1)\ \Delta d = \Delta d_{MAX} = \frac{[\text{BSA-TCO}_{26}]}{K_D + [\text{BSA-TCO}_{26}]}$$

Fig. S5 Equilibrium binding data of BSA–TCO_{26} to 40 nm citrate-capped gold nanoparticles. Data plotted as mean ± SD (N = 3), fitted (dashed line) to equation (1) to determine K_D.

Organic synthesis

Synthesis of methyltetrazine-OEG-amine

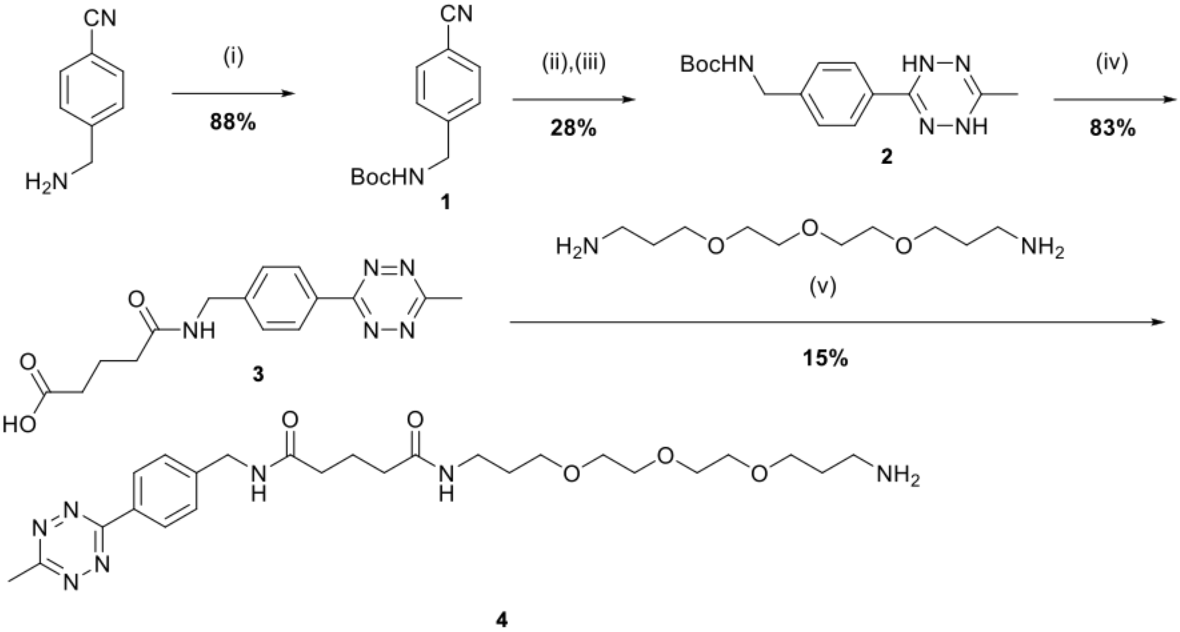

Fig. S6 *Reagents and conditions*: (i) Di-*tert*-butyl dicarbonate, NaOH, H_2O, 21 °C, 16 h. (ii) $Zn(OTf)_2$, 1,4-dioxane, MeCN, 65 °C, 72 h. (iii) $NaNO_2$, AcOH, DCM, 21 °C, 15 min. (iv) Glutaric anhydride, THF, 55 °C, 16 h. (v) Amine 70, NEt_3, HATU, DCM, 21 °C, 16 h.

tert-butyl (4-cyanobenzyl)carbamate **1**[2]

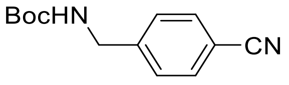

To 4-(aminomethyl)benzonitrile (5.0 g; 29.7 mmol) in H_2O (30 mL) was added to a stirring solution of NaOH (3.6 g, 89.1 mmol) and di-*tert*-butyl dicarbonate (7.1 g, 32.6 mmol) in H_2O (30 mL) at room temperature. The mixture was stirred for 16 h, after which time a white precipitate had formed. The mixture was filtered, washed with H_2O (100 mL), and the resulting solid dried under vacuum to yield compound **1** as a white solid (6.1 g, 26.2 mmol, 88%). ^1H NMR (400 MHz, $CDCl_3$) δ 7.62 (d, *J* = 8.3, 2H), 7.38 (d, *J* = 8.3, 2H), 4.96 (br s, 1H), 4.37 (d, *J* = 5.9 Hz, 2H), 1.46 (s, 9H); ^{13}C NMR (100 MHz, $CDCl_3$) δ 144.7 (C), 132.5 (C), 127.9 (C), 118.8 (C), 111.1 (C), 80.1 (C), 44.3 (CH_2), 28.4 (CH_3); IR (thin film) 3350, 2974, 2927, 2226, 1692 cm^{-1}.

tert-butyl (4-(6-methyl-1,2,4,5-tetrazin-3-yl)benzyl)carbamate **2**[2]

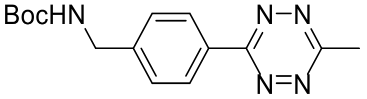

Hydrazine hydrate (80% w/w, 39.5 mL, 646 mmol) was added to a stirring suspension of tertbutylcarbamate **1** (3.0 g, 12.9 mmol), acetonitrile (6.72 mL, 12.9 mmol), and $Zn(OTf)_2$ (2.34 g, 6.46 mmol) in 1,4-dioxane (6 mL) at room temperature. The reaction was heated to 65 °C and stirred for 72 h. The reaction was cooled to room temperature and diluted with EtOAc (50 mL). The mixture was washed with 1 M HCl (50 mL) and the aqueous phase extracted with EtOAc (2 × 30 mL). The organic phase was dried ($MgSO_4$), filtered and evaporated under reduced pressure. The resulting crude residue was dissolved in a mixture of DCM and acetic acid (1:1, 200 mL), and $NaNO_2$ (17.8 g, 258 mmol) was added slowly over a period of 15 minutes, during which time the reaction turned bright red. The reaction was diluted with DCM (200 mL). The reaction mixture was washed with sodium bicarbonate (sat., aq., 200 mL) and the aqueous phase extracted with DCM (2 × 100 mL). The organic phase was dried ($MgSO_4$), filtered and the solvent removed *in vacuo*. The resulting residue was purified by column chromatography (20% EtOAc/petrol) to yield tetrazine **2** as a pink solid (1.07 g, 3.55 mmol, 28%). ^1H NMR (400 MHz, $CDCl_3$) δ 8.55 (d, *J* = 8.4 Hz, 2H), 7.5 (d, *J* = 8.3 Hz, 2H), 4.97 (br s, 1H), 4.44 (d, *J* = 5.8 hz, 2H), 3.09 (s, 3H), 1.48 (s, 9H); ^{13}C NMR (100 MHz, $CDCl_3$) δ 167.3 (C), 164.0 (C), 144.0 (C), 130.1 (C), 128.3 (C), 128.1 (C), 80.1 (C), 28.5 (CH_3), 21.1 (CH_3); IR (thin film) 3339, 2974, 2928, 1696, 1516 cm^{-1}.

5-((4-(6-methyl-1,2,4,5-tetrazin-3-yl)benzyl)amino)-5-oxopentanoic acid **3**[3]

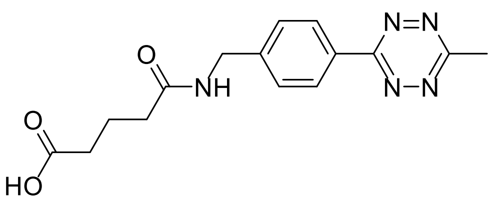

Tert-butyl (4-(6-methyl-1,2,4,5-tetrazin-3-yl)benzyl)carbamate **2** (800 mg, 2.65 mmol) in a mixture of TFA and DCM (1:4, 20 mL) was stirred at room temperature for 2 h. The solvent was removed *in vacuo* and the mixture re-dissolved in THF (50 mL). To this solution was added glutaric anhydride (605 mg, 5.31 mmol) and the mixture stirred at 55 °C for 16 h. The solvent was removed *in vacuo* and the mixture re-dissolved in saturated aqueous K_2CO_3 solution (100 mL). The mixture was then acidified with 15% HCl solution until the mixture stopped producing $CO_{2(g)}$ on acid addition. The mixture was then extracted with EtOAc (3 × 50 mL) and the combined organic phases washed with H_2O (4 × 30 mL), brine (30 mL), dried ($MgSO_4$). Any precipitate formed during extraction was re-dissolved in saturated aqueous K_2CO_3 solution (30 mL) and the work-up was repeated on this solution and the dried organic phases were combined, filtered and the solvent removed *in vacuo*, to yield compound **3** as a purple powder (691 mg, 2.2 mmol, 83%) without further purification. ^{1}H NMR (400 MHz, $CDCl_3$) δ 8.41 (d, *J* = 8.4, 2H), 7.51 (d, *J* = 8.5 Hz, 2H), 4.38 (d, *J* = 6.0 Hz, 2H), 2.98 (s, 3H), 4.27 (q, *J* = 7.4 Hz, 4H), 1.76 (p, *J* = 7.4 Hz, 2 H); ^{13}C NMR (100 MHz, $CDCl_3$) δ 174.2 (C), 171.9 (C), 167.1 (C), 163.2 (C), 144.5 (C), 130.4 (C), 128.0 (C), 127.5 (C), 41.9 (CH_2), 34.4 (CH_2), 33.0 (CH_2), 20.8 (CH_3), 20.7 (CH_2); IR (thin film) 3271, 3025, 2973, 2923, 2880, 1694, 1630, 1523 cm^{-1}.

N^1-(3-(2-(2-(3-aminopropoxy)ethoxy)ethoxy)propyl)-N^5-(4-(6-methyl-1,2,4,5-tetrazin-3-yl)benzyl)glutaramide **4**[4]

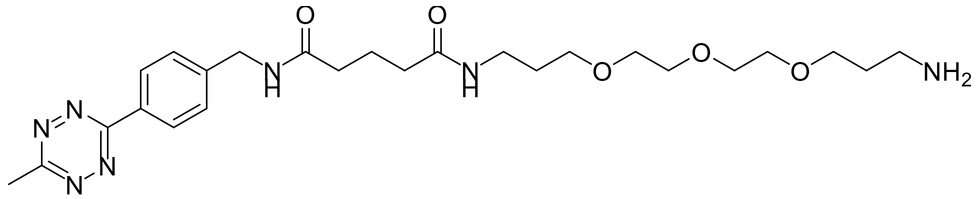

To a solution of *O,O'*-bis(3-aminopropyl)diethylene glycol (280 mg, 1.27 mmol) in DCM (5 mL) was added slowly, at room temperature, a solution of 5-((4-(6-methyl-1,2,4,5-tetrazin-3-yl)benzyl)amino)-5-oxopentanoic acid **3** (200 mg, 0.63 mmol), HATU (240 mg, 0.63 mmol), and NEt_3 (64 mg, 0.63 mmol) in DCM (5 mL). The resulting solution was stirred at room temperature for 16 h. The solvent was removed *in vacuo*, and the mixture re-dissolved in 1 M HCl solution (20 mL) and washed with DCM (3 × 20 mL) to remove unreacted 5-((4-(6-methyl-1,2,4,5-tetrazin-3-yl)benzyl)amino)-5-oxopentanoic acid **7**. The aqueous phase was then basified with saturated aqueous K_2CO_3 solution, extracted with DCM (3 × 20 mL). The combined organic phases were extracted with 1 M HCl solution (20 mL). The aqueous phase was basified with saturated aqueous K_2CO_3 solution and extracted with DCM (3 × 20 mL). The combined organic phases were washed with brine (20 mL), dried ($MgSO_4$), filtered and the solvent removed *in vacuo*. The crude residue was purified by flash column chromatography (10–30% MeOH in DCM) to afford compound **4** (47.6 mg, 15%) as a purple oil. ^{1}H NMR (600 MHz, $CDCl_3$) δ 8.49 (d, *J* = 8.4 Hz, 2H), 8.35 (t, *J* = 5.8 Hz, 1H), 7.57 (d, *J* = 8.5 Hz, 2H), 6.67 (t, *J* = 6.1 Hz, 1H), 4.52 (d, *J* = 6.2 Hz, 2H), 3.71 (t, *J* = 5.3 Hz, 2H), 3.65 – 3.61 (m, 4H), 3.61 – 3.57 (m, 4H), 3.50 (t, *J* = 5.6 Hz, 2H), 3.32 (q, *J* = 6.8 Hz, 2H), 3.19 – 3.15 (m, 2H), 3.08 (s, 3H), 2.40 (t, *J* = 7.5 Hz, 2H), 2.30 (t, *J* = 6.9 Hz, 2H), 2.03 – 1.96 (m, 4H), 1.77 (p, *J* = 6 Hz, 2H); ^{13}C NMR (150 MHz, $CDCl_3$) δ 174.0 (C), 173.3 (C), 167.2 (C), 164.2 (C), 144.6 (C), 130.4 (C), 128.8 (CH), 128.1 (CH), 70.9 (CH_2), 70.7 (CH_2), 69.8 (CH_2), 69.7 (CH_2), 69.6 (CH_2), 68.3 (CH_2), 43.0 (CH_2), 40.7 (CH_2), 36.5 (CH_2), 35.4 (CH_2), 35.1 (CH_2), 30.0 (CH_2), 26.1 (CH_2), 21.9 (CH_2), 21.3 (CH_3). IR (thin film) 3302, 2945, 2831, 1642, 1630, 1542 cm^{-1}.

Synthesis of methyltetrazine-diBrPD

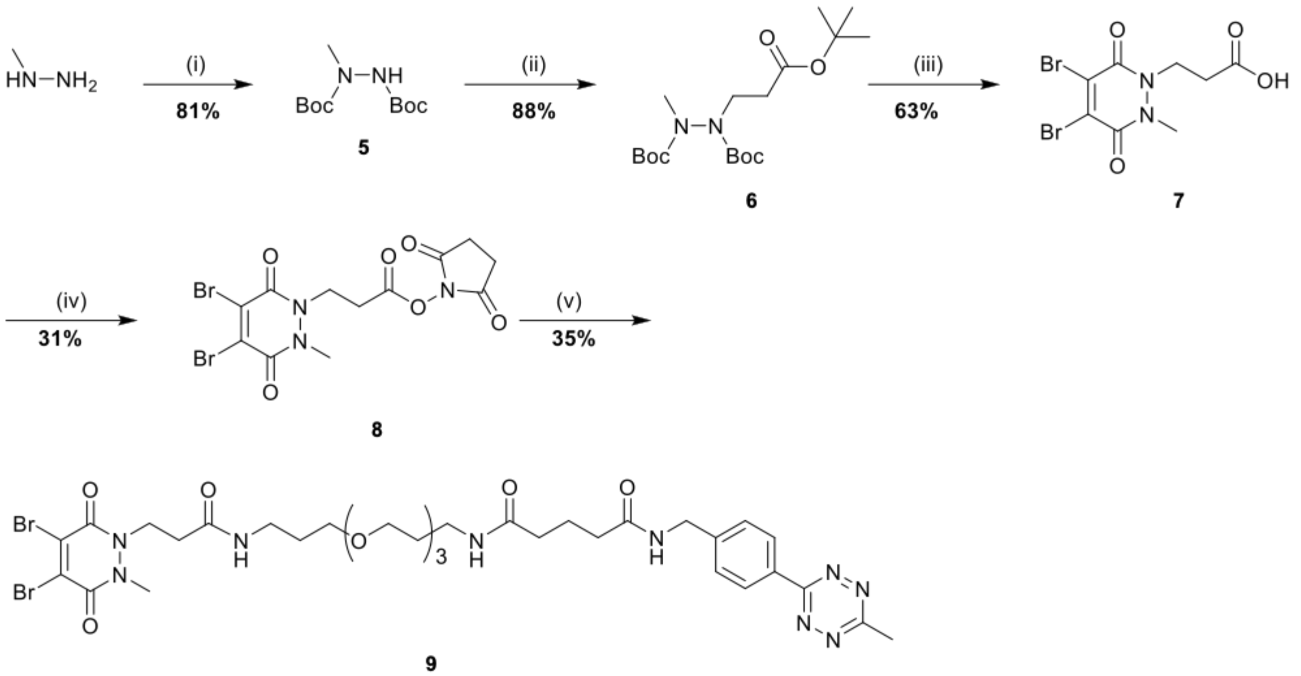

Fig S7 Synthesis of methyltetrazine–diBrPD 9. *Reagents and conditions*: (i) Di-*tert*-butyl decarbonate, propan-2-ol, DCM, 21 °C, 16 h. (ii) *tert*-butanol, 10% $NaOH_{(aq)}$, *tert*-butyl acrylate, 60 °C, 24 h. (iii) Dibromomaleic anhydride, AcOH, reflux, 4 h. (iv) DCC, NHS, dry THF, 21 °C, 16 h. (v) Amine **4**, NEt_3, DCM, 21 °C, 3 h.

di-*tert*-butyl 1-methylhydrazine-1,2-dicarboxylate **5**[5]

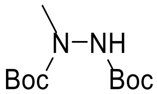

To a solution of methyl hydrazine (1.14 mL, 21.7 mmol) in propan-2-ol (16 mL), was added a solution of di-*tert*-butyl dicarbonate (11.4 g, 52.1 mmol, pre-dissolved in CH_2Cl_2 (12 mL)) drop-wise over 30 min. The reaction was then stirred at 21 °C for 16 h. After this time, the solvents were then removed *in vacuo* and the crude residue purified by flash column chromatography (0% to 15% EtOAc/petrol) to afford di-*tert*-butyl-1-methylhydrazine-1,2-dicarboxylate **5** (4.67 g, 19.1 mmol, 88%) as a white solid. ^{1}H NMR (400 MHz, CDCl3, rotamers) δ 6.41–6.15 (m, 1H) 3.10 (s, 3H), 1.46–1.45 (m, 18H); ^{13}C NMR (150 MHz, $CDCl_3$, rotamers) δ 81.3 (C), 37.5 (CH_3), 28.3 (CH_3); IR (solid) 3299, 2974, 2929, 1703 cm^{-1}.

di-*tert*-butyl 1-(3-(*tert*-butoxy)-3-oxopropyl)-2-methylhydrazine-1,2-dicarboxylate **6**[5]

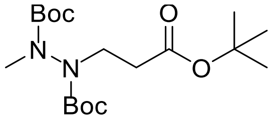

To a solution of di-*tert*-butyl 1-methylhydrazine-1,2-dicarboxylate **5** (3.00 g, 12.2 mmol) in *tert*-butanol (15 mL) was added 10% NaOH (0.5 mL) and the reaction mixture stirred at 21 °C for 10 min. After this time, *tert*-butyl acrylate (5.31 mL, 36.6 mmol) was added to the solution and the reaction mixture was heated at 60 °C for 24 h. Following this, the solvent was removed *in vacuo* and the crude residue was dissolved in EtOAc (150 mL) and washed with water (3 × 50 mL). The organic layer was then dried ($MgSO_4$) and concentrated *in vacuo*. Purification of the crude residue by flash column chromatography (0% to 20% EtOAc/petrol) afforded di-*tert*-butyl-1-(3-(*tert*-butoxy)-3-oxopropyl)-2- methylhydrazine-1,2-dicarboxylate **6** (3.33 g, 8.91 mmol, 73%) as a clear oil. ^{1}H NMR (600 MHz, $CDCl_3$, rotamers) δ 3.82–3.47 (m, 2H), 3.03–2.94 (m, 3H), 2.47 (t, *J* = 7.1 Hz, 2H), 1.48–1.37 (m, 27H); ^{13}C NMR (150 MHz, $CDCl_3$, rotamers) δ 171.0 (C), 155.4 (C), 154.4 (C), 81.0 (C), 44.6 (CH_3), 36.6 (CH_2), 34.1 (CH_2), 28.3 (CH_3); IR (thin film) 2974, 2931, 1709 cm^{-1}.

3-(4,5-dibromo-2-methyl-3,6-dioxo-3,6-dihydropyridazin-1(2*H*)-yl)propanoic acid **7**

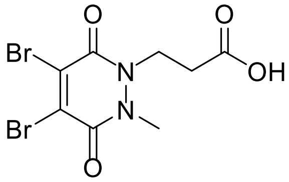

Dibromomaleic anhydride (4.00 g; 14.61 mmol) was dissolved in AcOH (80 mL) and heated under reflux for 30 min. To this solution was added di-*tert*-butyl 1-(3-(*tert*-butoxy)-3-oxopropyl)-2-methylhydrazine-1,2-dicarboxylate **6** (4.77 g; 12.75 mmol) and the reaction heated under reflux for a further 4 h. The solvent was

removed *in vacuo* by co-evaporation with toluene and the mixture column purified (50-100% EtOAc 1% AcOH/petrol) to yield 3-(4,5-dibromo-2-methyl-3,6-dioxo-3,6-dihydropyridazin-1(2*H*)-yl)propanoic acid **7** (3.22 g; 71%) as a yellow powder. ^{1}H NMR (700 MHZ, $CDCl_3$) δ 4.41 (t, *J* = 7.1 Hz, 2H), 3.69 (s, 3H), 2.77 (t, *J* = 7.1 Hz, 2H); ^{13}C NMR (150 MHz, $CDCl_3$) δ 173.9 (C), 153.5 (C), 153.2 (C), 136.4 (C), 135.7 (C), 43.5 (CH_2), 35.3 (CH_3), 31.2 (CH_2); IR (thin film) 3300-2700, 2924, 1726, 1617, 1570, 1442, 1396 cm^{-1}.

2,5-dioxopyrrolidin-1-yl 3-(4,5-dibromo-2-methyl-3,6-dioxo-3,6-dihydropyridazin-1(2*H*)-yl)propanoate **8**[5]

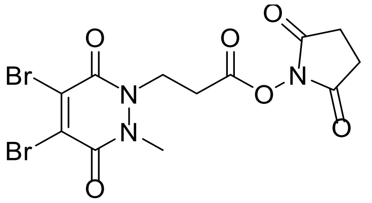

To a solution of 3-(4,5-dibromo-2-methyl-3,6-dioxo-3,6-dihydropyridazin-1(2*H*)-yl)propanoic acid **7** (2.00 g; 5.62 mmol) in dry THF (40 mL) at 0 °C was added DCC (1.28 g; 6.2 mmol). The solution was stirred at 0 °C for 30 min. Following this, was added NHS (718 mg, 6.2 mmol) and the reaction stirred at room temperature for a further 16 h. The solvent was removed *in vacuo* and the crude residue purified by column chromatography (20-100% EtOAc/petrol) to yield pyridazinedione **8** (796 mg, 1.76 mmol, 31 %) as a white powder. ^{1}H NMR (700 MHZ, $CDCl_3$) δ 4.48 (t, J= 6.9, 2H), 3.68 (s, 3H), 3.10 (t, *J* = 6.9, 2H), 2.85 (br, 4H); ^{13}C NMR (175 MHz, $CDCl_3$) δ 168.7 (C), 166.0 (C), 153.3 (C), 153.1 (C), 136.9 (C), 135.5 (C), 43.5 (CH_2), 35.3 (CH_3), 30.46 (CH_2), 25.7 (CH_2); IR (thin film) 2927, 2851, 1733, 1632, 1571, 1203 cm^{-1}.

N1-(17-(4,5-dibromo-2-methyl-3,6-dioxo-3,6-dihydropyridazin-1*(2H)-yl*)-15-oxo-4,7,10-trioxa-14-azaheptadecyl)-N5-(4-(6-methyl-1,2,4,5-tetrazin-3-yl)benzyl)glutaramide **9**[4]

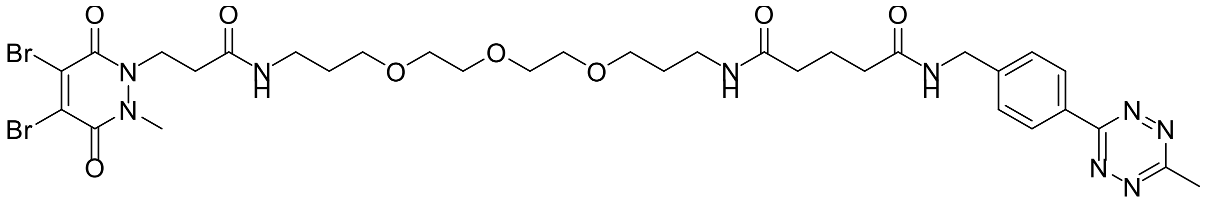

To a solution of 2,5-dioxopyrrolidin-1-yl 3-(4,5-dibromo-2-methyl-3,6-dioxo-3,6-dihydropyridazin-1(2*H*)-yl) propanoate **8** (61.2 mg, 0.14 mmol) in DCM (5 mL), was added N^{1}-(3-(2-(2-(3-aminopropoxy)ethoxy)ethoxy)propyl)-N^{5}-(4-(6-methyl-1,2,4,5-tetrazin-3-yl)benzyl)glutaramide **4** (27.6 mg, 0.058 mmol) and NEt_3 (7.4 μL, 5.4 mg, 0.053 mmol) and the reaction mixture was stirred at rt for 3 h. After this time, the reaction was concentrated *in vacuo* and the crude residue dissolved in $CHCl_3$ (25 mL) and washed with water (2 × 15 mL) and saturated aq. K_2CO_3 (15 mL). The organic layer was then dried ($MgSO_4$) and concentrated *in vacuo.* Purification of the crude residue by flash column chromatography (5% to 20% MeOH/EtOAc) afforded compound **9** (15.8 mg, 0.018 mmol, 32%) as a purple oil. ^{1}H NMR (600 MHz, $CDCl_3$) δ 8.53 (d, *J* = 8.4 Hz, 2H), 7.50 (d, *J* = 8.5 Hz, 2H), 7.04 (t, *J* = 5.2 Hz, 1H), 6.80 (t, *J* = 5.8 Hz, 1H), 6.46 (t, *J* = 5.3 Hz, 1H), 4.55 (d, *J* = 6.0 Hz, 2H), 4.39 (t, *J* = 6.9 Hz, 2H), 3.69 (s, 3H), 3.63 – 3.50 (m, 12H), 3.36 – 3.26 (m, 4H), 3.09 (s, 3H), 2.80 (d, *J* = 4.8 Hz, 1H), 2.56 (t, *J* = 6.9 Hz, 2H), 2.36 (t, *J* = 7.3 Hz, 2H), 2.26 (t, *J* = 6.9 Hz, 2H), 1.99 (app. p, *J* = 7.1 Hz, 2H), 1.78 – 1.69 (m, 4H); ^{13}C NMR (150 MHz, $CDCl_3$) δ 173.0 (C), 172.8 (C), 169.2 (C), 167.4 (C), 164.0 (C), 153.1 (C), 152.9 (C), 143.6 (C), 136.4 (C), 135.4 (C), 131.0 (C), 128.6 (2×CH), 128.3 (2×CH), 70.6 (CH_2), 70.1 (CH_2), 70.0 (2×CH_2), 70.0 (2×CH_2), 44.6 (CH_2), 43.3 (CH_2), 38.2 (CH_2), 38.0 (CH_2), 35.6 (CH_2), 35.4 (CH_2), 35.2 (CH_3), 34.1 (CH_2), 29.1 (CH_2), 28.9 (CH_2), 22.2 (CH_2), 21.3 (CH_3); IR (thin film) 3310, 2923, 2851, 1734, 1631, 1543 cm^{-1}; LRMS (ESI). 858 (50, $[M^{81}Br^{81}Br+H]^+$), 856 (100, $[M^{79}Br^{81}Br+H]^+$), 854 (50, $[M^{79}Br^{79}Br+H]^+$); HRMS (ESI) calcd for $C_{33}H_{46}Br_2N_9O_8$ $[M^{79}Br^{81}Br+Na]^+$ 878.1630; observed 878.1639.

NMR spectra of chemical compounds

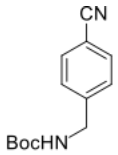

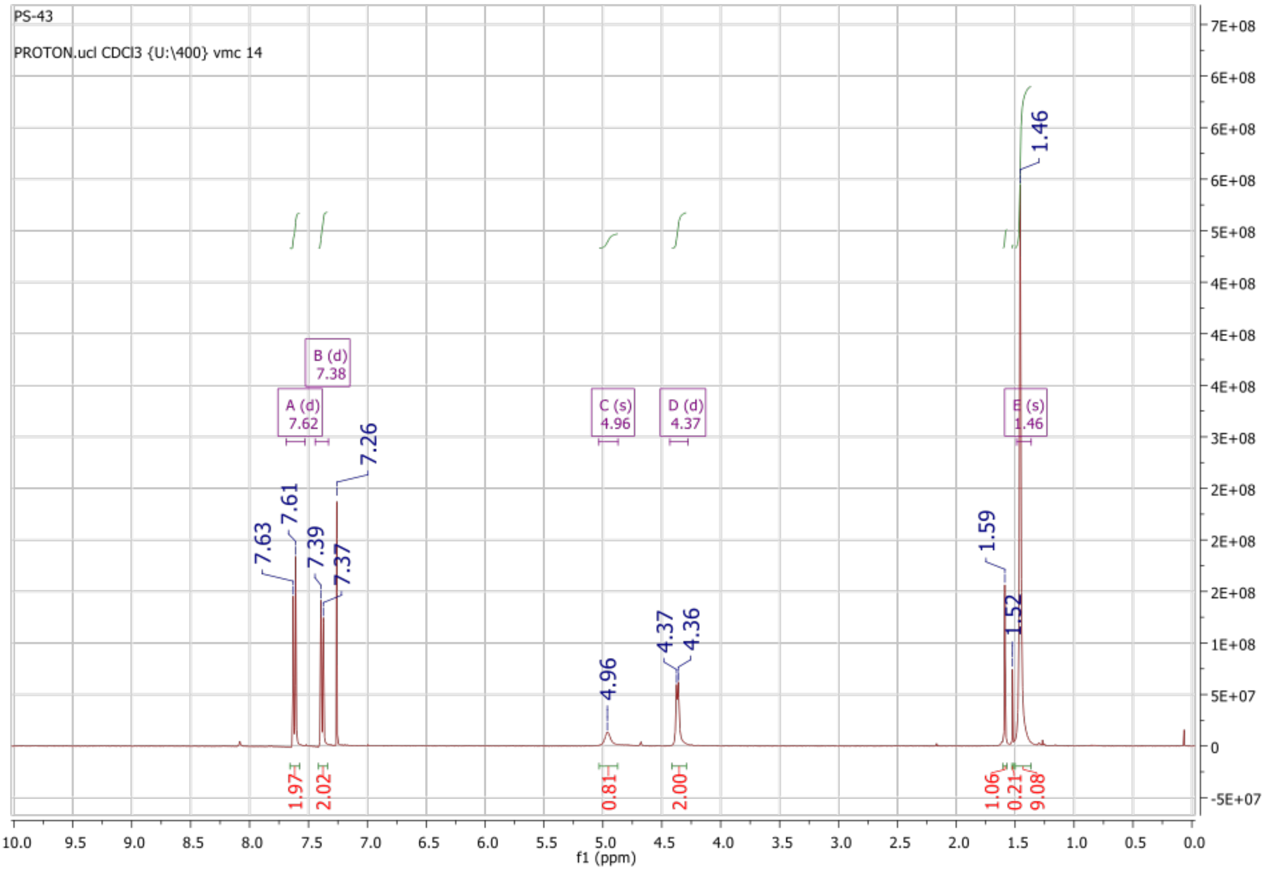

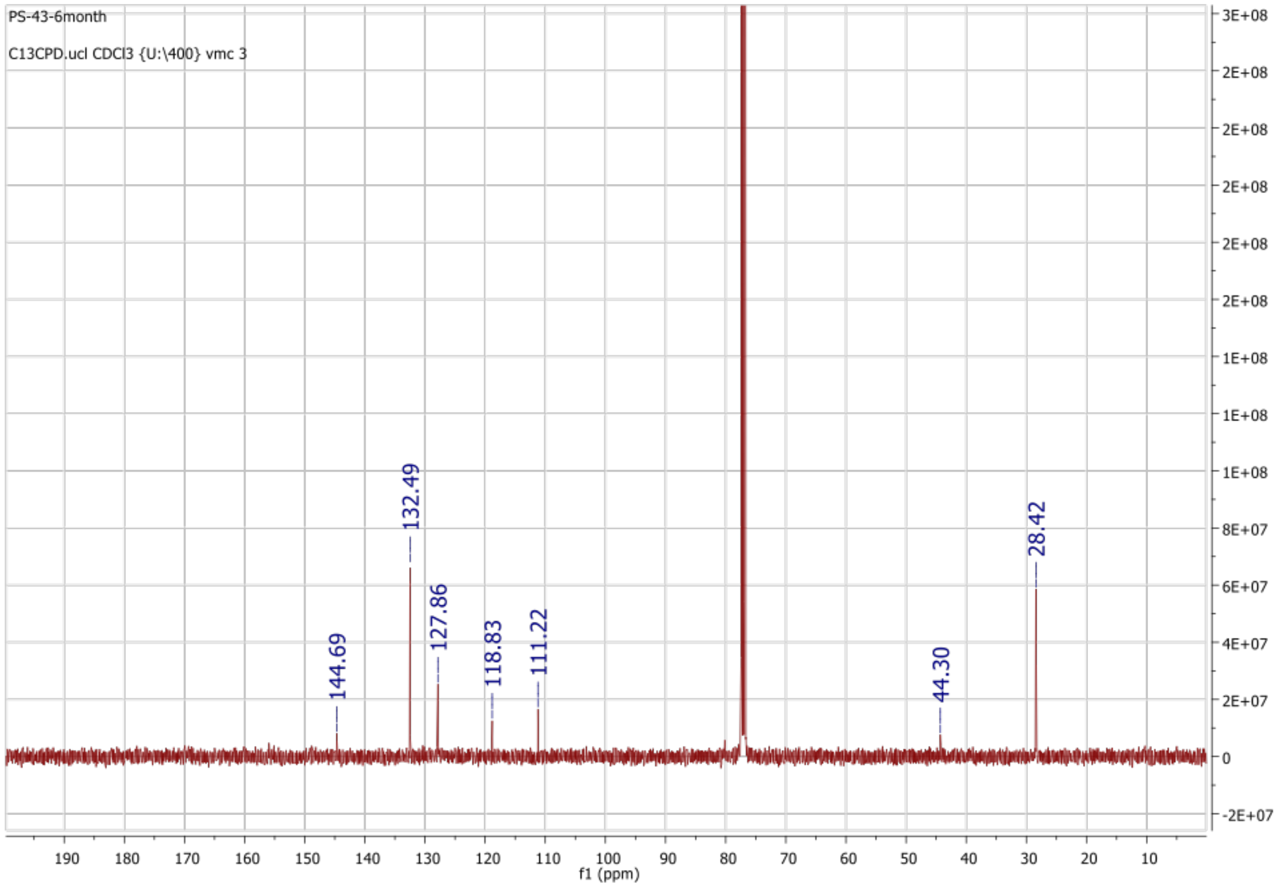

Fig. S8 ^{1}H and ^{13}C spectra for *tert*-butyl (4-cyanobenzyl)carbamate **1**.

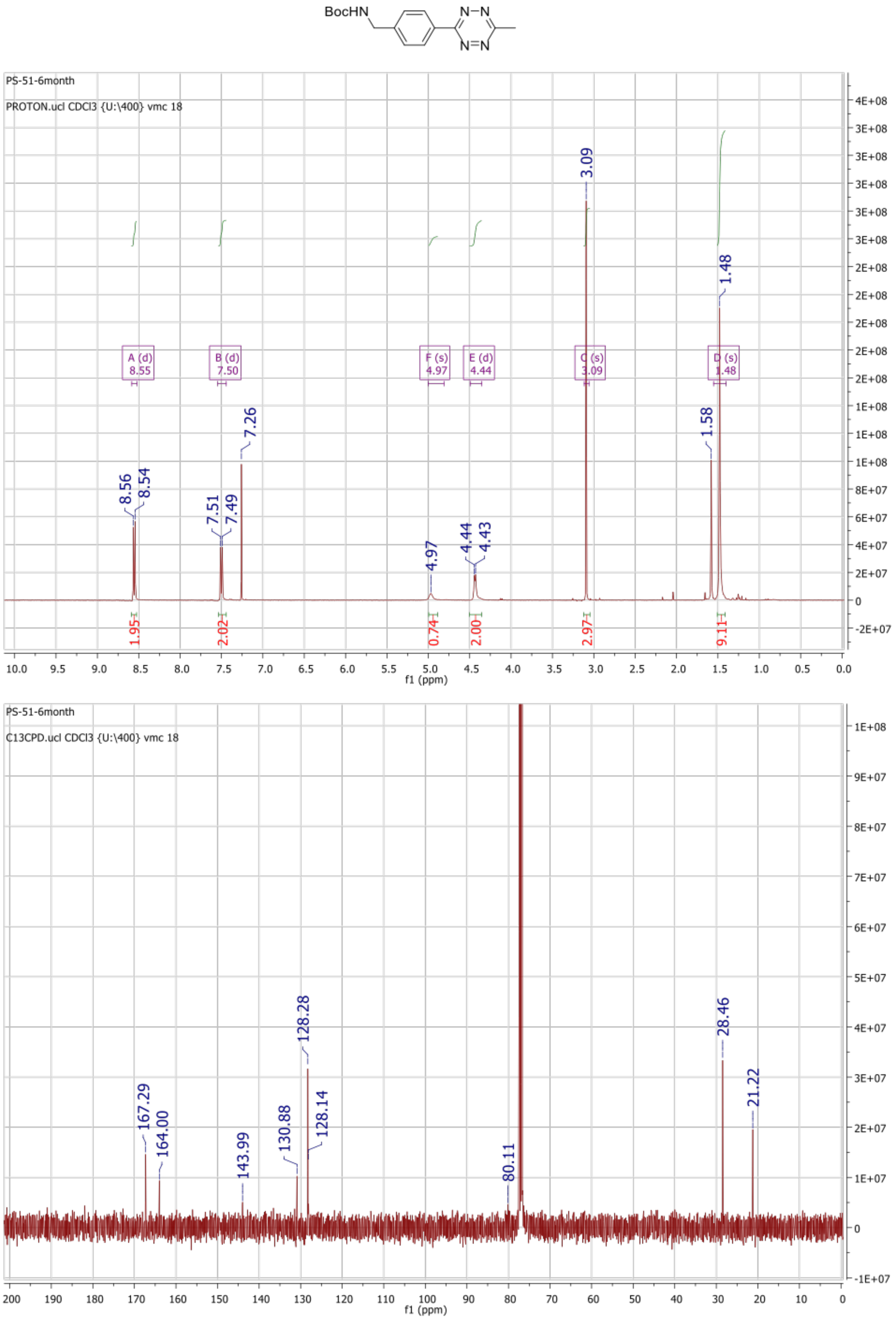

Fig. S9 ^{1}H and ^{13}C spectra for t*ert*-butyl (4-(6-methyl-1,2,4,5-tetrazin-3-yl)benzyl)carbamate **2**.

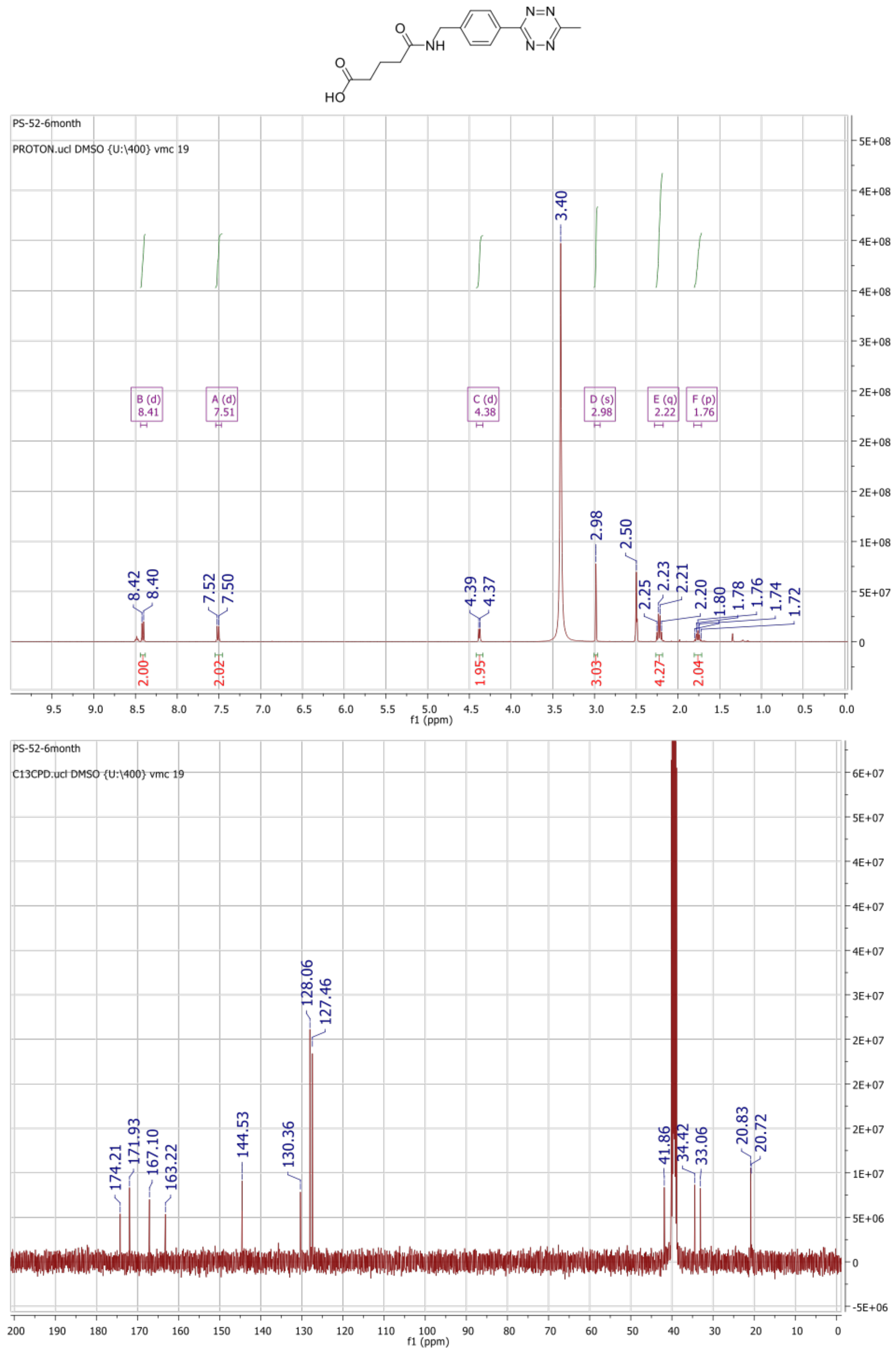

Fig. S10 ^{1}H and ^{13}C spectra for 5-((4-(6-methyl-1,2,4,5-tetrazin-3-yl)benzyl)amino)-5-oxopentanoic acid **3**.

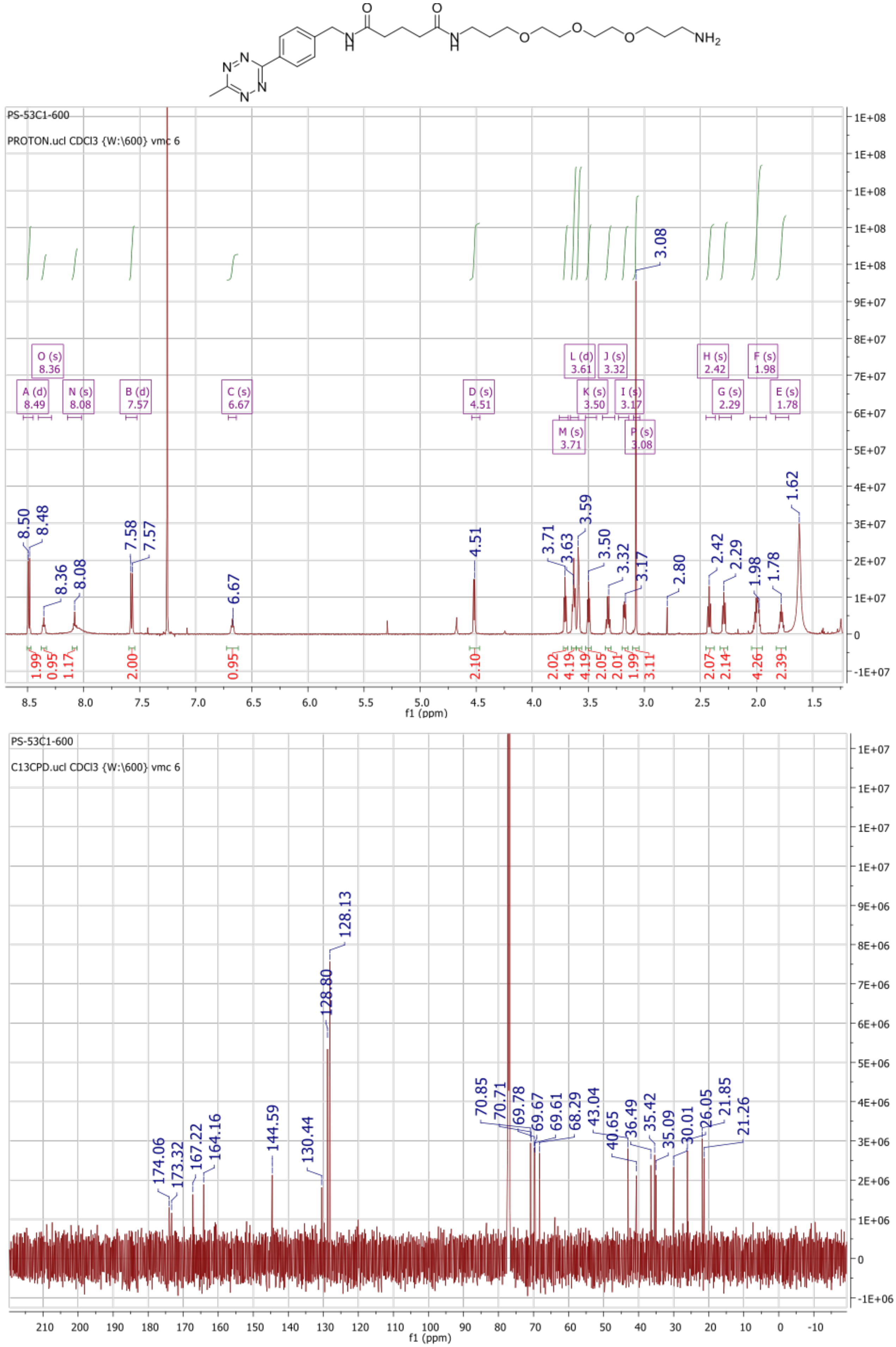

Fig. S11 ^{1}H and ^{13}C spectra for N^{1}-(3-(2-(2-(3-aminopropoxy)ethoxy)ethoxy)propyl)-N^{5}-(4-(6-methyl-1,2,4,5-tetrazin-3-yl)benzyl)glutaramide **4**.

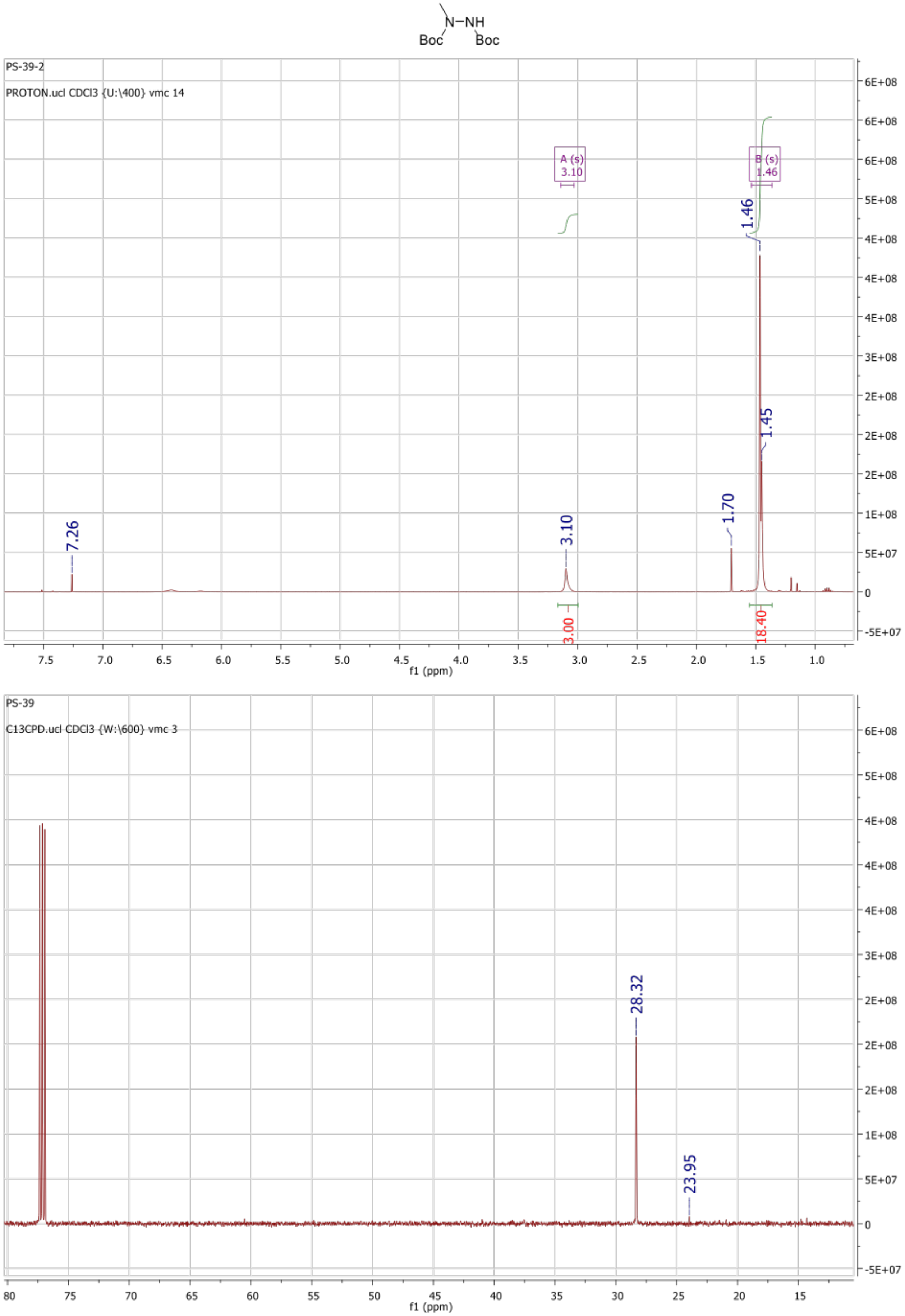

Fig. S12 ^{1}H and ^{13}C spectra for di-*tert*-butyl 1-methylhydrazine-1,2-dicarboxylate **5**.

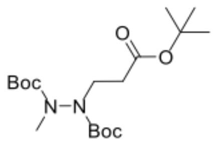

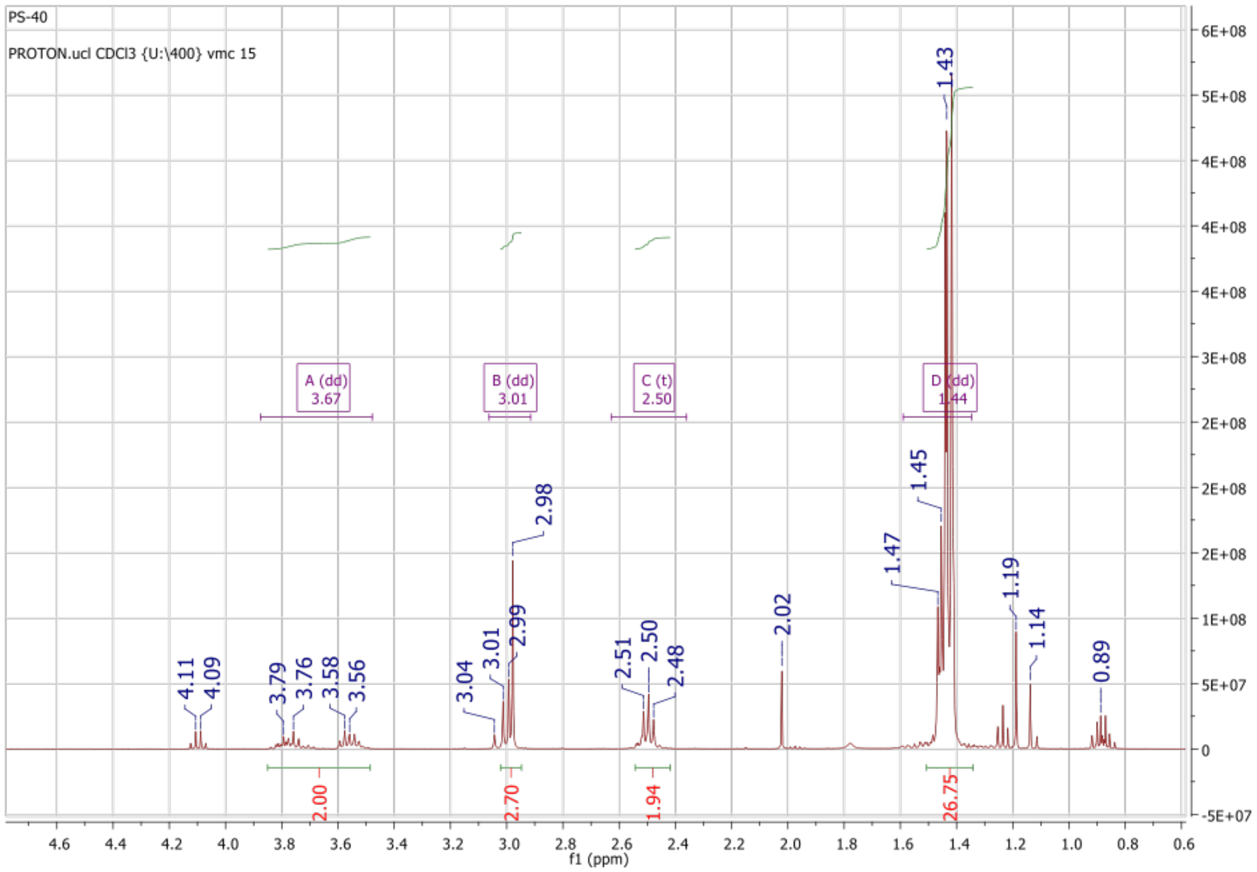

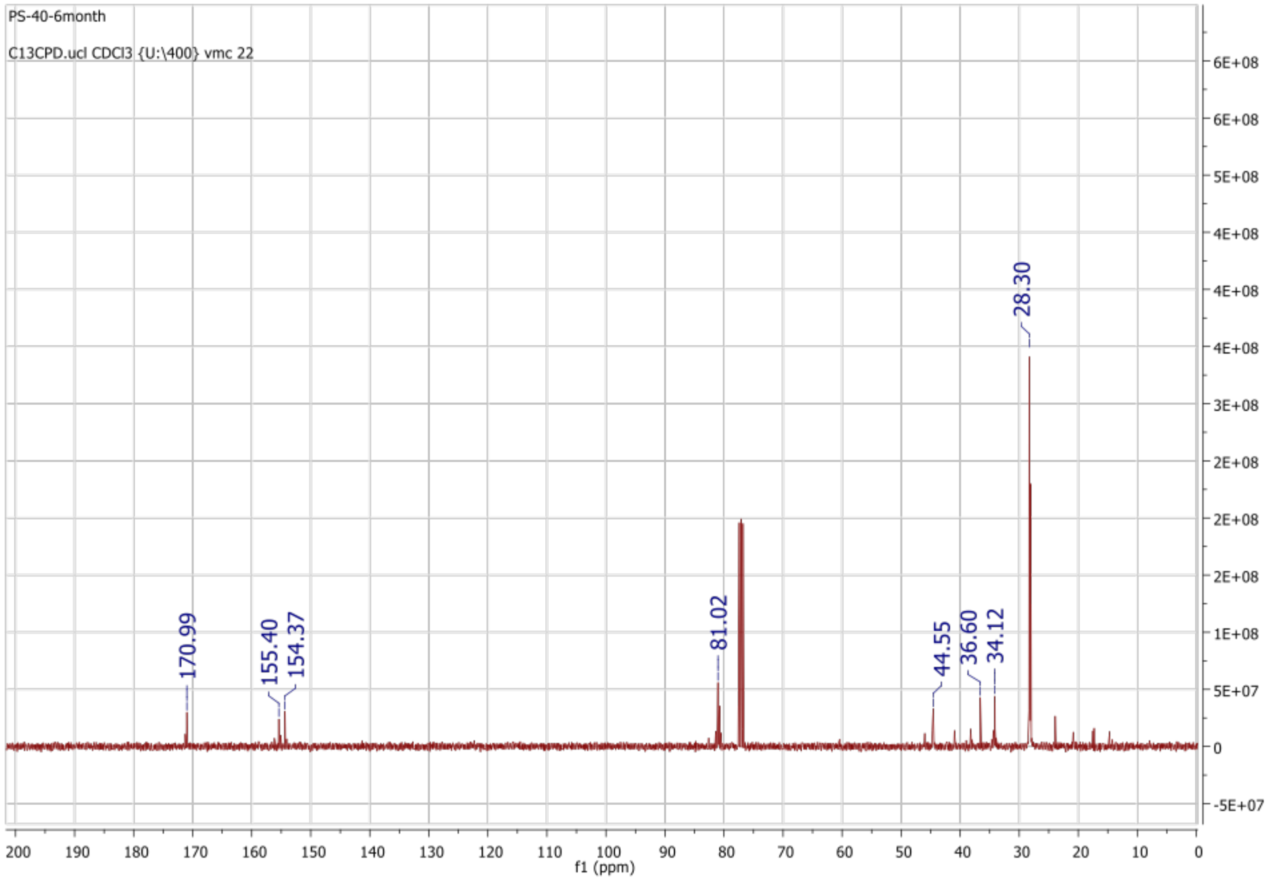

Fig. S13 ^{1}H and ^{13}C spectra for di-*tert*-butyl 1-(3-(*tert*-butoxy)-3-oxopropyl)-2-methylhydrazine-1,2-dicarboxylate **6**.

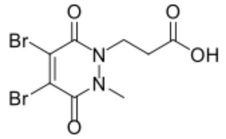

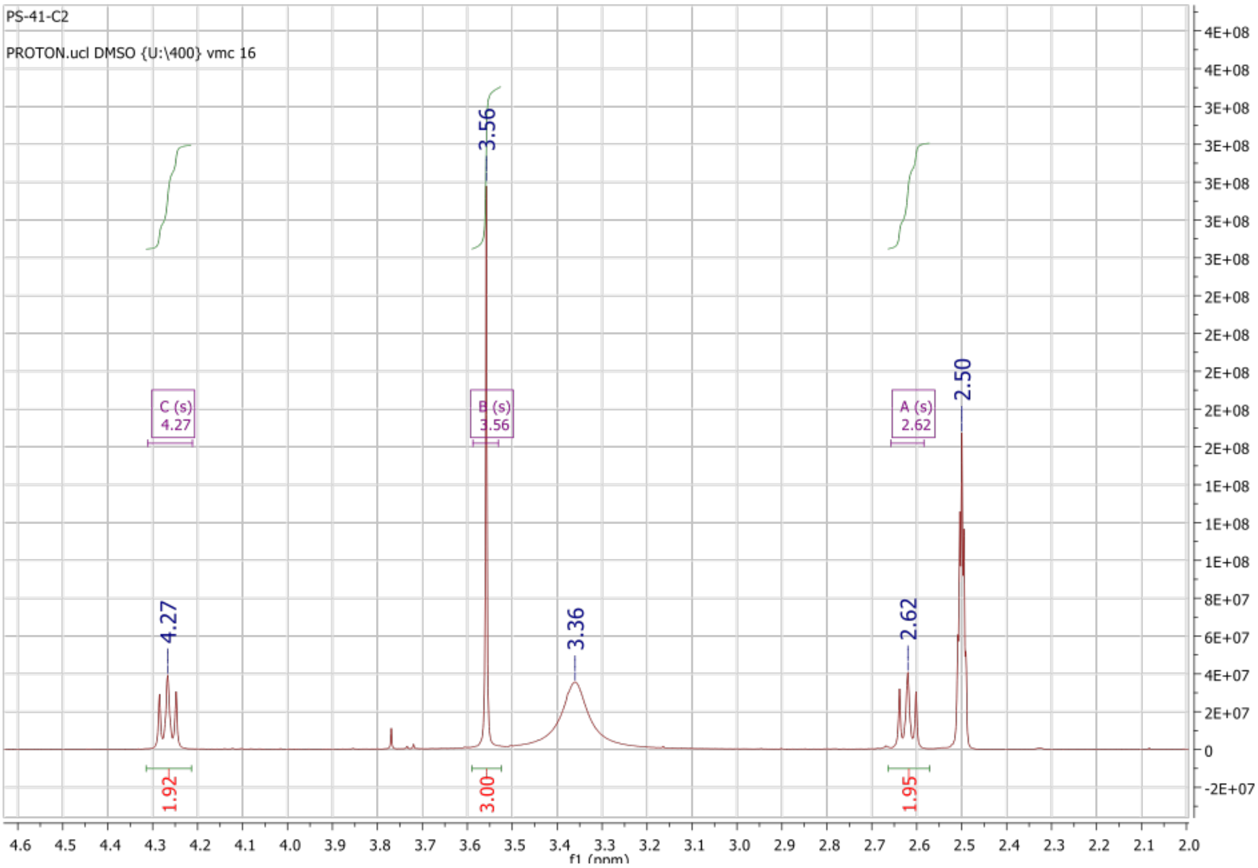

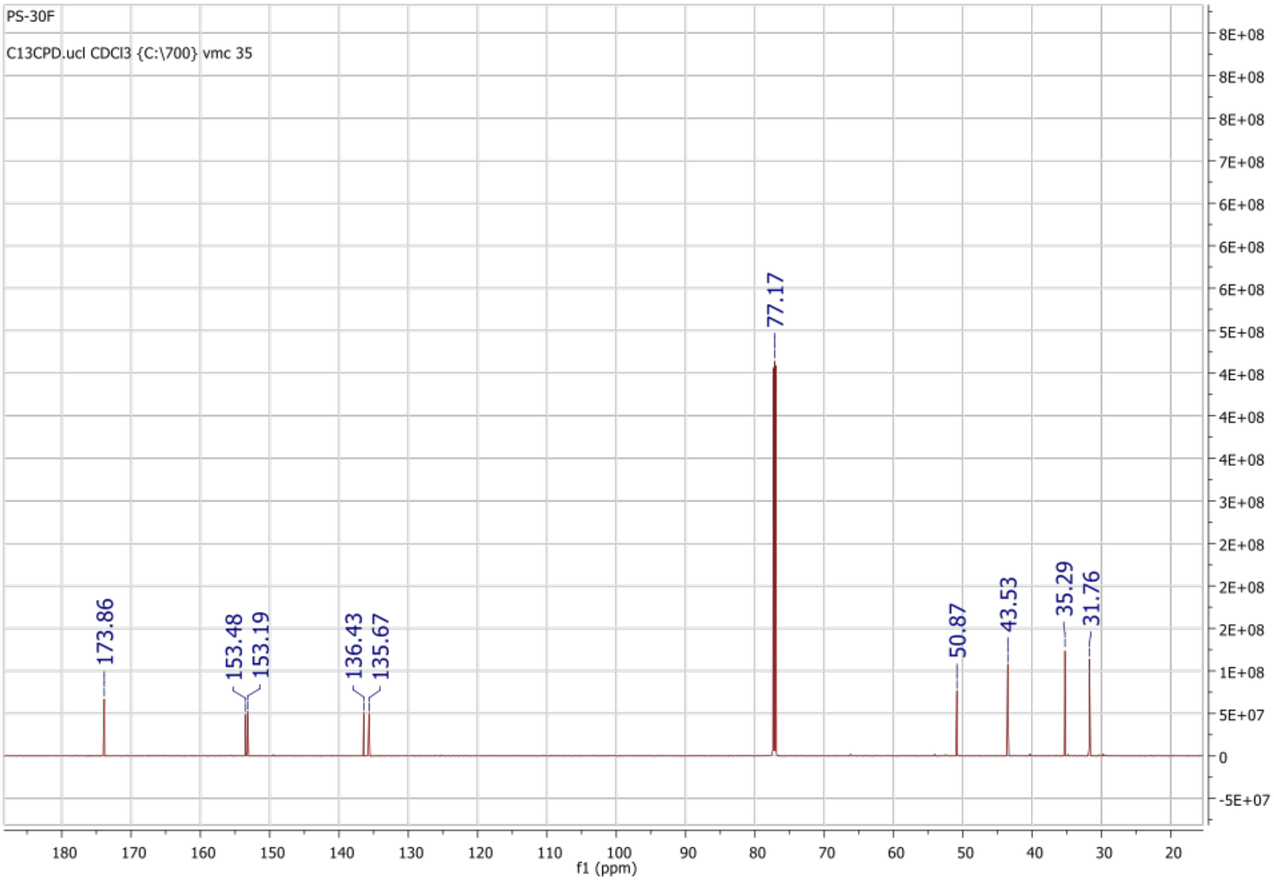

Fig. S14 ^{1}H and ^{13}C spectra for 3-(4,5-dibromo-2-methyl-3,6-dioxo-3,6-dihydropyridazin-1*(2H)-yl*)propanoic acid **7**.

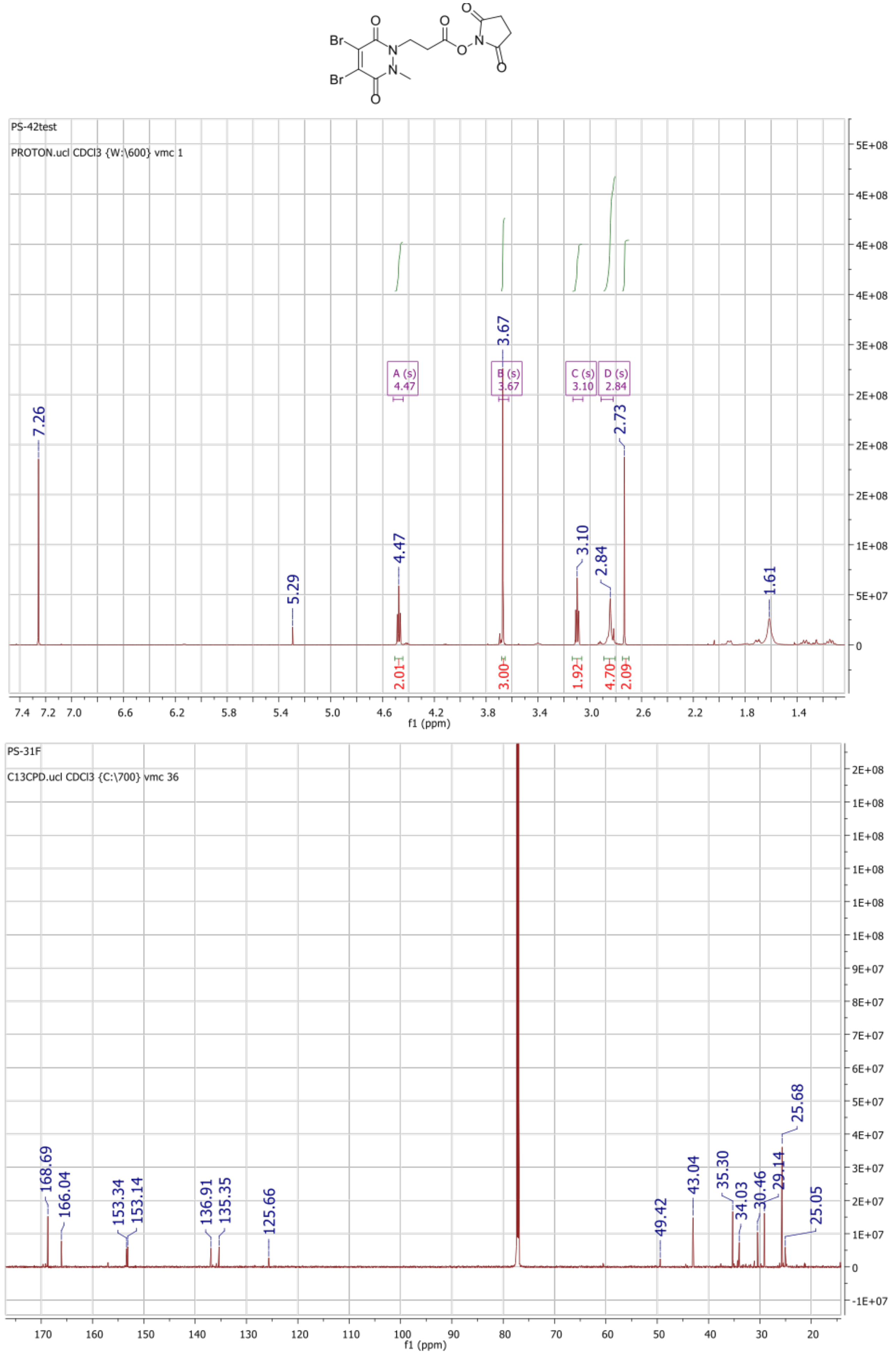

Fig. S15 ^{1}H and ^{13}C spectra for 2,5-dioxopyrrolidin-1-yl 3-(4,5-dibromo-2-methyl-3,6-dioxo-3,6-dihydropyridazin-1*(2H)-yl)*propanoate **8**.

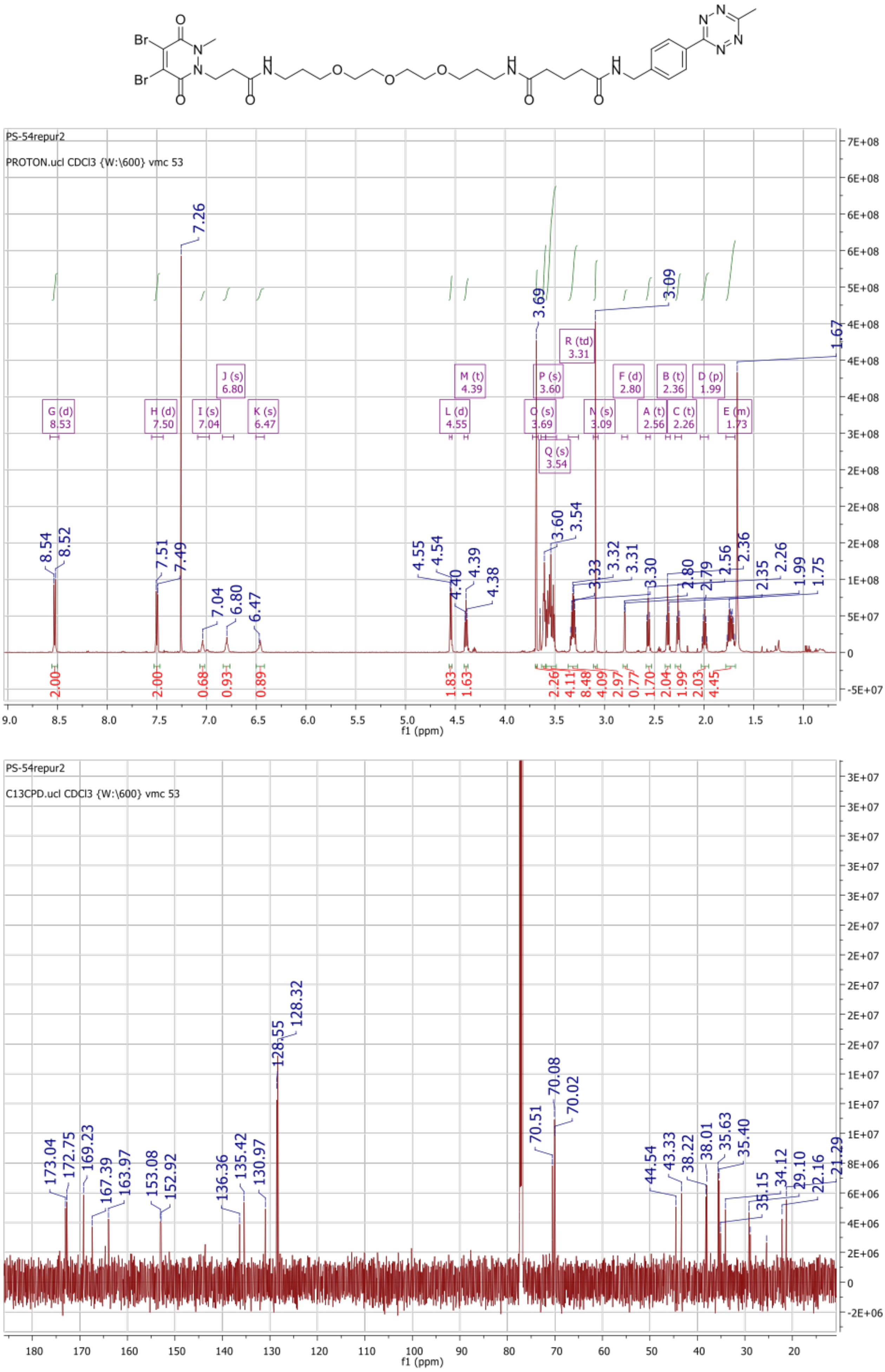

Fig. S16 ^{1}H and ^{13}C spectra for N^1-(17-(4,5-dibromo-2-methyl-3,6-dioxo-3,6-dihydropyridazin-1*(2H)-yl*)-15-oxo-4,7,10-trioxa-14-azaheptadecyl)-N^5-(4-(6-methyl-1,2,4,5-tetrazin-3-yl)benzyl)glutaramide **9**.

Antibody modification

To a solution of Ontruzant (50 μL, 20 μM, BBS–EDTA pH = 8.0), tris(2-carboxyethyl)phosphine hydrochloride (50 equiv., 2.5 μL, 20 mM in BBS pH = 8.0) was added and the solution incubated at 37 °C for 1 hour. The protein was subsequently purified *via* ultrafiltration (10,000 Da MWCO, 6 × 2000 μL) into BBS–EDTA pH = 8.0, and the concentration adjusted to 20 μM. diBrPD–Tet **9** (3.0 μL, 60 equiv., 20 mM in DMSO) was added and the solution incubated at 21 °C for 6 hours. The protein was subsequently purified *via* ultrafiltration (10,000 Da MWCO, 6 × 2000 μL) into d.d. H_2O, the concentration quantified, and used directly. UV-Vis spectroscopy was used to determine protein concentrations and pyridazinedione to antibody ratios (PDAR). Buffer was used as blank for baseline correction with extinction coefficients; ε_{280} = 215,000 M^{-1} cm^{-1} for Ontruzant and ε_{335} = 9,100 M^{-1} cm^{-1} for pyridazinedione scaffolds. A correction factor at 280 nm of 0.25 (of ε_{335}) was employed for pyridazinedione scaffolds. The equation below describes the calculation of the concentration of Ontruzant.

$$c = \frac{A}{\varepsilon_{280}}$$

The equation system below describes the calculation of the PDAR (*n*) for Ontruzant-PD conjugates.

$$A_{280} = (\varepsilon_{280} + (0.25 \times n \times \varepsilon_{335})) \times c$$

and

$$A_{335} = n \times \varepsilon_{335} \times c$$

Thus:

$$n = \frac{\varepsilon_{280} \times A_{335}}{A_{280} \times \varepsilon_{335} + (0.25 \times A_{335} \times \varepsilon_{335})}$$

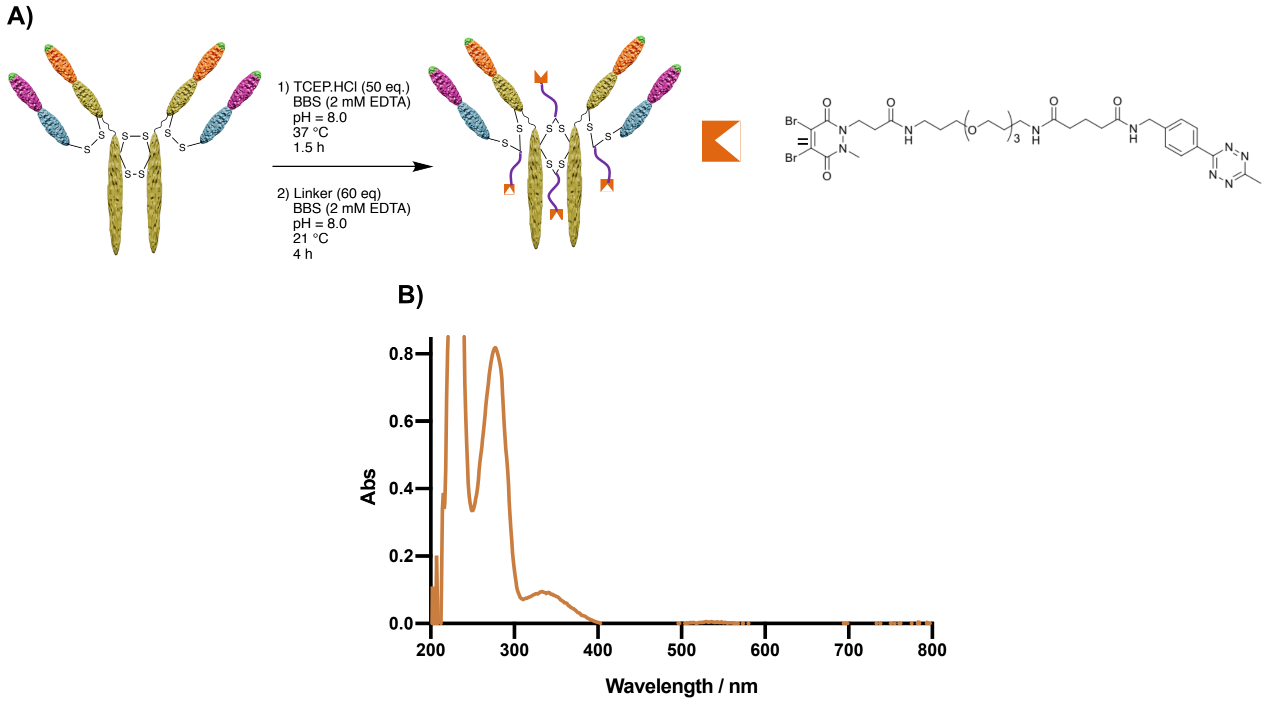

Fig. S17 Modification of ONT with methyltetrazine-diBrPD **9**. (A) Schematic representation of the modification of Ontruzant (ONT) with methyltetrazine–diBrPD **9**. (B) UV-Vis spectra of ONT–Tet, with the pyridazinedione (PD) chromophore clearly observed at 335 nm.

pH optimisation

BSA–TCO_{26}–ONT–AuNP pH optimisation
Solutions of BSA–TCO_{26}–AuNPs (108 μL, OD = 1.1) were prepared in triplicate in the following buffers; 20 mM phosphate buffer pH = 6.0, 7.0, 8.0, and 20 mM carbonate buffer pH = 9.0, 10.0. All buffers contained 0.05% tween 20. To these solutions ONT (12 μL, 300 nM in d.d. H_2O) was added and the samples incubated at 21 °C for 16 hours. The particle solutions were subsequently centrifuged (5000 r.c.f, 10 minutes) to pellet the particles, and then washed (6 x 1 mL carbonate storage buffer). After the final wash the particles were resuspended into carbonate storage buffer, briefly vortexed and sonicated, and stored at 4 °C. Binding to HER2 was assessed using a sandwich LFIA as detailed in the methods section.

ONT–AuNP pH optimisation
Solutions of ONT (213 μL, 0.59 μM in BBS pH = 8.0 – 10.0) were prepared in duplicate and added to sample tubes. To each sample, 40 nm citrate-capped AuNPs (50 μL, OD = 5, BBS solutions) were added and the solutions left to incubate at 21 °C for 1 hour, before the UV-Vis spectra were obtained on a plate reader.

Concentration optimisation

BSA–TCO_{26}–ONT–AuNP concentration optimisation
To a solution of BSA–TCO_{26}–AuNPs (135 μL, OD = 1.1 in storage buffer), ONT–Tet (15 μL, 125 – 8000 nM in d.d H_2O) was added and the solution left to incubate at 21 °C for 16 hours. The particle solution was subsequently centrifuged (5000 r.c.f, 10 minutes) to pellet the particles, and then washed (6 x 1 mL carbonate storage buffer). After the final wash the particles were resuspended into carbonate storage buffer, briefly vortexed and sonicated, and stored at 4 °C. Binding to HER2 was assessed using a sandwich LFIA as detailed in the methods section.

ONT–AuNP concentration optimisation
To a solution of 40 nm citrate AuNPs (135 μL, OD = 1.1 in pH = 7.0 phosphate buffer), ONT (15 μL, 125 – 8000 nM in d.d H_2O) was added and the solution left to incubate at 21 °C for 16 hours. Blocking buffer (50 μL) was added and the solution incubated at 21 °C for 30 minutes. The particle solution was subsequently centrifuged (5000 r.c.f, 10 minutes) to pellet the particles, and then washed (6 x 1 mL carbonate storage buffer). After the final wash the particles were resuspended into carbonate storage buffer, briefly vortexed and sonicated, and stored at 4 °C. Binding to HER2 was assessed using a sandwich LFIA as detailed in the methods section.

Time optimisation

BSA–TCO_{26}–ONT–AuNP time optimisation
To a solution of BSA–TCO_{26}–AuNPs (135 μL, OD = 1.1 in storage buffer), ONT–Tet (15 μL, 400 nM in d.d H_2O) was added and the solution left to incubate at 21 °C for 1 – 32 hours. The particle solution was subsequently centrifuged (5000 r.c.f, 10 minutes) to pellet the particles, and then washed (6 x 1 mL carbonate storage buffer). After the final wash the particles were resuspended into carbonate storage buffer, briefly vortexed and sonicated, and stored at 4 °C. Binding to HER2 was assessed using a sandwich LFIA as detailed in the methods section.

ONT–AuNP time optimisation
To a solution of 40 nm citrate AuNPs (135 μL, OD = 1.1 in pH = 7.0 phosphate buffer), ONT (15 μL, 400 nM in d.d H_2O) was added and the solution left to incubate at 21 °C for 1 – 32 hours. Blocking buffer (50 μL) was added and the solution incubated at 21 °C for 30 minutes. The particle solution was subsequently centrifuged (5000 r.c.f, 10 minutes) to pellet the particles, and then washed (6 x 1 mL carbonate storage buffer). After the final wash the particles were resuspended into carbonate storage buffer, briefly vortexed and sonicated, and stored at 4 °C. Binding to HER2 was assessed using a sandwich LFIA as detailed in the methods section

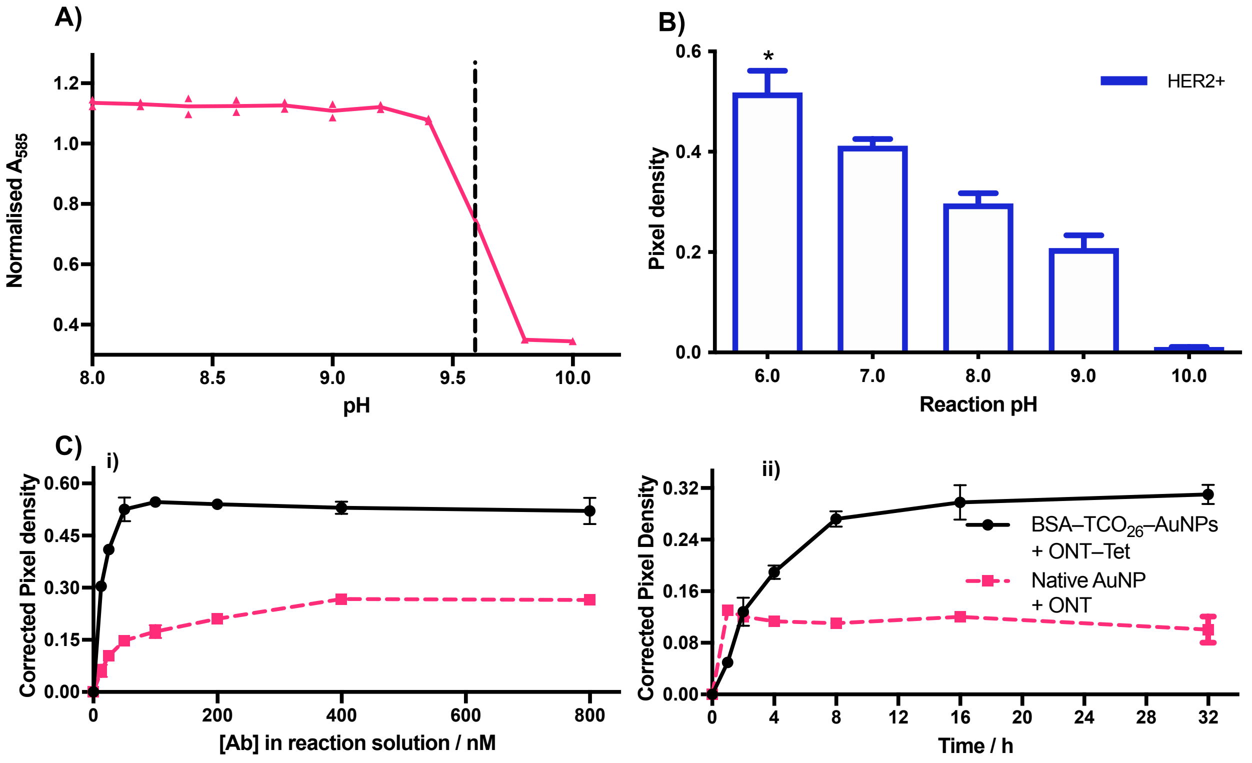

Fig. S18 Optimisation of the pH of attachment of ONT and ONT–Tet to Native AuNP and BSA–TCO_{26}–AuNPs, respectively. (A) A flocculation curve showing A_{585} *vs* pH for the physisorption of ONT to citrate capped 40 nm AuNPs. Individual data points are plotted, with a solid line representing the mean (N = 2). Dotted line indicates the point of flocculation. (B) Sandwich LFIA data showing the binding of BSA–TCO_{26}–ONT AuNPs to HER2 to determine the optimal pH for construction of these particles. * indicates the presence of aggregation. Data plotted as mean ± SD (N = 3). No signal was observed in HER2 negative controls. C) HER2 binding data (LFIA) as a function of (i) antibody concentration in solution, (ii) time. As a comparison physisorption of native ONT to 40 nm AuNPs was also included (dotted magenta line). Data plotted as mean ± SD (N = 3).

Antibody per particle quantification

To a solution of BSA–TCO_{26}–AuNPs (280 μL, 0.166 nM, OD = 1.1111, phosphate buffer pH = 7.0 0.05% tween 20), ONT–Tet (20 μL, 600 nM, d.d. H_2O) was added. A control solution of the same volume and concentration of particles was prepared, and d.d. H_2O was added in the place of ONT–Tet. A standard curve (in triplicate) of ONT–Tet (0 – 150 nM, phosphate buffer pH = 7.0 0.05% tween 20) was also prepared. Each solution was incubated at 21 °C for 16 hours. The samples were centrifuged (5000 r.c.f, 10 minutes), and 100 μL of the supernatant from each sample and standard was added to a black 96 well plate. To each sample and standard, a working CBQCA reagent solution (0.4 mM ATTO-TAG CBQCA, 1.33 mM KCN, Invitrogen™) was added. The plate was covered and incubated at 21 °C for 1 hour with constant shaking (500 rpm). The fluorescence emission was read (λ_{ex}/ λ_{ex} = 465 / 550, SpectraMax M5, Molecular Devices). The controls were subtracted, and the antibody concentration in the supernatant was determined from interpolation of the standard curve. The number of IgG ligands per particle was determined according to the following equation:

$$(2)\ \text{No. IgGs} = \frac{[\text{ONT}]_S - [\text{ONT}]_E}{[\text{AuNP}]_S}$$

Where $[\text{ONT}]_S$ is the concentration of ONT added to the reaction, $[\text{ONT}]_E$ is the concentration of ONT at the end of the reaction, as determined from the standard curve, and [AuNP] is the concentration of AuNPs added to the reaction (0.15 nM). In the case of iEDDA click reaction, ONT represents ONT–Tet.

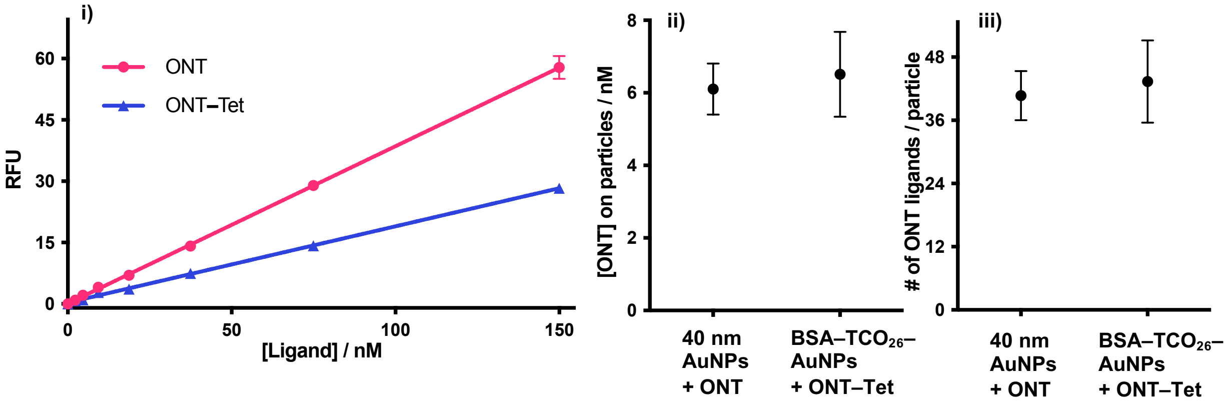

Fig. S19 Quantification of the number of ONT and ONT–Tet ligands attached to Native AuNP and BSA–TCO26–AuNPs, respectively. (i) Calibration curves for CBQCA assays performed on ONT and ONT–Tet. (ii) Determination of the concentration of ONT or ONT–Tet attached to Native AuNP and BSA–TCO26–AuNPs, respectively. (iii) Number of ligands per nanoparticle determined according to equation (2). All data plotted as mean ± SD (N = 3).

Nanoparticle stability test

Serial dilutions of BSA and GSH were prepared (150 μL, 2.0 ×10^{-3} – 2000 μM in storage buffer) in Eppendorf tubes. To these solutions, BSA–Biotin–AuNPs were added (300 μL), and the mixtures incubated at 21 °C for 5 hours. The particles were pelleted via centrifugation (5000 r.c.f, 10 minutes), and the supernatant from each sample was removed. The concentration of BSA–Biotin in the supernatant was deduced using ELISA. Briefly, a NeutraAvidin coated 96 well plate (Pierce) was blocked with 2% BSA in PBST (0.1% tween 20) for 1 hour at 21 °C, and then washed with PBST. The Supernatant (100 μL) from each sample was added to the plate, along with a standard curve of BSA–Biotin (0.137 – 100 nM in storage buffer), which was incubated at 21 °C for 90 minutes followed by washing with PBST. Streptavidin–HRP (100 μL, 1 in 80,000 dilution in PBST + 2% BSA, Abcam) was added to the plate, which was incubated at 21 °C for 90 minutes followed by washing with PBST. Visualization of the formed complex was achieved using OneStep Ultra TMB (Thermo Scientific™) according to the manufacturer’s instructions. The plate was read at 450 nm using a SpectraMax M5 plate reader (Molecular Devices).

Lateral flow strips

These images are the raw data for the LFIA plot in Fig. 7 of the manuscript.

Physisorbed ONT AuNPs

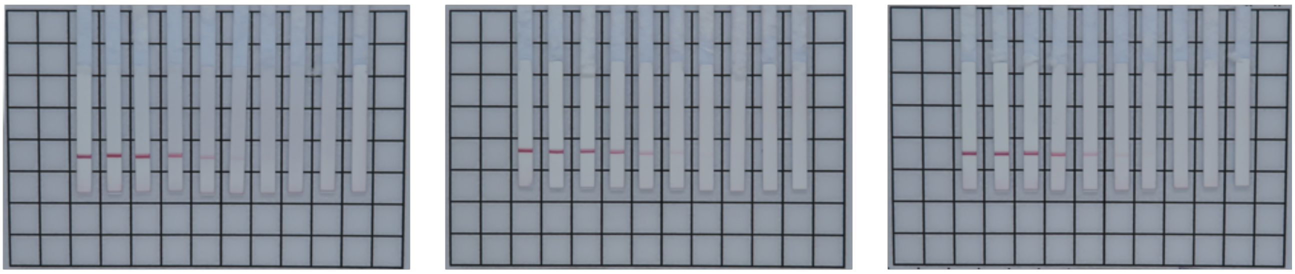

BSA–TCO_{25}–ONT

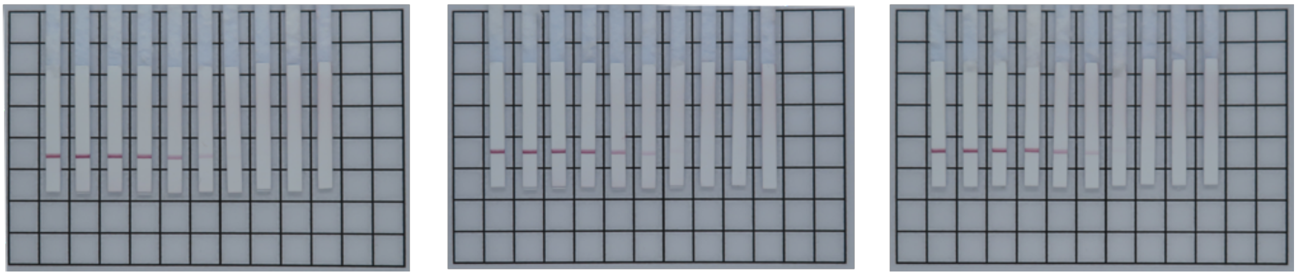

Fig. S20 Raw lateral flow test images for the HER2 LFIA. The top three panels are the experimental repeats for the LFIA performed using AuNP–ONT conjugates (prepared using physisorption). The bottom three panels are the experimental repeats for the LFIA performed using BSA–TCO_{25}–ONT–AuNPs.

References

(1) Ma, W. et al. Modular Assembly of Proteins on Nanoparticles. *Nat. Commun.* **2018**, *9* (1), 1–9.

(2) Lang, K. et al. Genetic Encoding of Bicyclononynes and Trans-Cyclooctenes for Site-Specific Protein Labeling in Vitro and in Live Mammalian Cells via Rapid Fluorogenic Diels-Alder Reactions. *J. Am. Chem. Soc.* **2012**, *134* (25), 10317–10320.

(3) Hernández-Gil, J. et al. Development of 68Ga-Labelled Ultrasound Microbubbles for Whole-Body PET Imaging. *Chem. Sci.* **2019**, *10* (21), 5603–5615.

(4) Maruani, A. et al. A Plug-and-Play Approach for the de Novo Generation of Dually Functionalized Bispecifics. *Bioconjug. Chem.* **2020**, *31* (3), 520–529.

(5) Bahou, C. et al. Highly Homogeneous Antibody Modification through Optimisation of the Synthesis and Conjugation of Functionalised Dibromopyridazinediones. *Org. Biomol. Chem.* **2018**, *16*, 1359–1366.
